# Acute Endotoxin-Induced Thymic Atrophy Is Characterized By Intrathymic Inflammatory and Wound Healing Responses

**DOI:** 10.1371/journal.pone.0017940

**Published:** 2011-03-18

**Authors:** Matthew J. Billard, Amanda L. Gruver, Gregory D. Sempowski

**Affiliations:** 1 Department of Biostatistics & Bioinformatics, Duke University Medical Center, Durham, North Carolina, United States of America; 2 Department of Medicine, Department of Pathology, and the Duke University Human Vaccine Institute, Duke University Medical Center, Durham, North Carolina, United States of America; New York University, United States of America

## Abstract

**Background:**

Productive thymopoiesis is essential for a robust and healthy immune system. Thymus unfortunately is acutely sensitive to stress resulting in involution and decreased T cell production. Thymic involution is a complication of many clinical settings, including infection, malnutrition, starvation, and irradiation or immunosuppressive therapies. Systemic rises in glucocorticoids and inflammatory cytokines are known to contribute to thymic atrophy. Little is known, however, about intrathymic mechanisms that may actively contribute to thymus atrophy or initiate thymic recovery following stress events.

**Methodology/Principal Findings:**

Phenotypic, histologic and transcriptome/pathway analysis of murine thymic tissue during the early stages of endotoxemia-induced thymic involution was performed to identify putative mechanisms that drive thymic involution during stress. Thymus atrophy in this murine model was confirmed by down-regulation of genes involved in T cell development, cell activation, and cell cycle progression, correlating with observed phenotypic and histologic thymus involution. Significant gene changes support the hypothesis that multiple key intrathymic pathways are differentially activated during stress-induced thymic involution. These included direct activation of thymus tissue by LPS through TLR signaling, local expression of inflammatory cytokines, inhibition of T cell signaling, and induction of wound healing/tissue remodeling.

**Conclusions/Significance:**

Taken together, these observations demonstrated that in addition to the classic systemic response, a direct intrathymic response to endotoxin challenge concurrently contributes to thymic involution during endotoxemia. These findings are a substantial advancement over current understanding of thymus response to stress and may lead to the development of novel therapeutic approaches to ameliorate immune deficiency associated with stress events.

## Introduction

Ongoing and productive thymopoiesis is essential for development and maintenance of a robust and healthy immune system. Many factors have a negative effect on thymopoiesis and acute thymic atrophy is a complication among a variety of clinical settings, including bacterial infection [1], starvation [2], and irradiation or immunosuppressive therapies [3]. In response to stress, thymus tissue involutes and output of naïve T cells is substantially reduced, leaving the host potentially vulnerable to new infections. The systemic response to stress has been well-described and involves a rise in glucocorticoids and pro-inflammatory cytokines; which we and others have shown negatively impact thymopoiesis and contribute greatly to thymus atrophy [1], [4]. Little is known, however, about intrathymic mechanisms that may actively drive thymus involution in response to stress. In contrast to chronic thymic involution associated with aging, acute stress-induced thymic atrophy can resolve naturally over time after the stressor is removed [5]. By defining intrathymic mechanisms driving thymic atrophy and tissue recovery, potential therapies could be developed to help accelerate thymic recovery and immune reconstitution.

Endotoxemia-induced acute thymic atrophy is a useful model to study the impact of acute stress on thymopoiesis [5]. Acute thymic atrophy in response to endotoxin stress is characterized by specific loss of CD4+CD8+ double positive (DP) thymocytes and a dramatic reduction in T cell development [1], [6]. Thymic architecture is severely perturbed following endotoxin challenge, with damage to thymus epithelium and visible remodeling of thymic morphology, involving loss of distinct cortico-medullary junctions [7], [8]. Activation of the hypothalamus-pituitary-adrenal (HPA) axis, resulting in a systemic rise in glucocorticoids and an acute systemic pro-inflammatory cytokine cascade, contributes to acute thymic involution [4]. Important systemic pro-inflammatory cytokines include tumor necrosis factor (TNF), interleukin-1 (IL-1), and IL-6 [9]. Inflammation activates nuclear factor-kappa B (NF-κB), a crucial signaling molecule in the immune response [10] and thymocyte development [11]. While it is clear that these systemic mechanisms play an important role in stress-induced acute thymic atrophy [12], underlying intrathymic mechanisms are now also being appreciated as contributing factors. Previous work from this laboratory has demonstrated that the IL-6 family member, leukemia inhibitory factor (LIF), mediates acute thymic atrophy locally by stimulation of intrathymic pathways and systemically by activation of the HPA axis [13]. Furthermore, in a model of age-related, chronic thymic involution, investigators compared rapidly-involuting strains of mice with slowly-involuting strains of the same age. These studies identified lowered IL-7 and anti-apoptotic BCL-2, and elevated pro-apoptotic BAD gene expression levels, as contributors to rapid involution and disruption of thymopoiesis [14].

These reports support an evolving paradigm of thymus involution, in which intrathymic mechanisms actively contribute to increased local inflammation, decreased thymus output, and increased tissue atrophy. It is therefore hypothesized that intrathymic pathways are active during endotoxemia-induced acute thymic atrophy leading to thymopoietic and morphologic dysregulation. A phenotypic, histologic and transcriptome/pathway analysis of murine thymic tissue during the early stages of endotoxemia-induced involution was undertaken in this study to define intrathymic mechanisms that drive acute thymic atrophy. Reported findings support the hypothesis that multiple key intrathymic pathways are differentially activated during endotoxemia-induced thymic involution. These include direct activation of thymus tissue by LPS through TLR signaling, local expression of inflammatory cytokines, inhibition of T cell signaling, and induction of wound healing/tissue remodeling.

## Results

### Acute Thymic Atrophy Induced by Endotoxin Challenge

**Figure 1:**
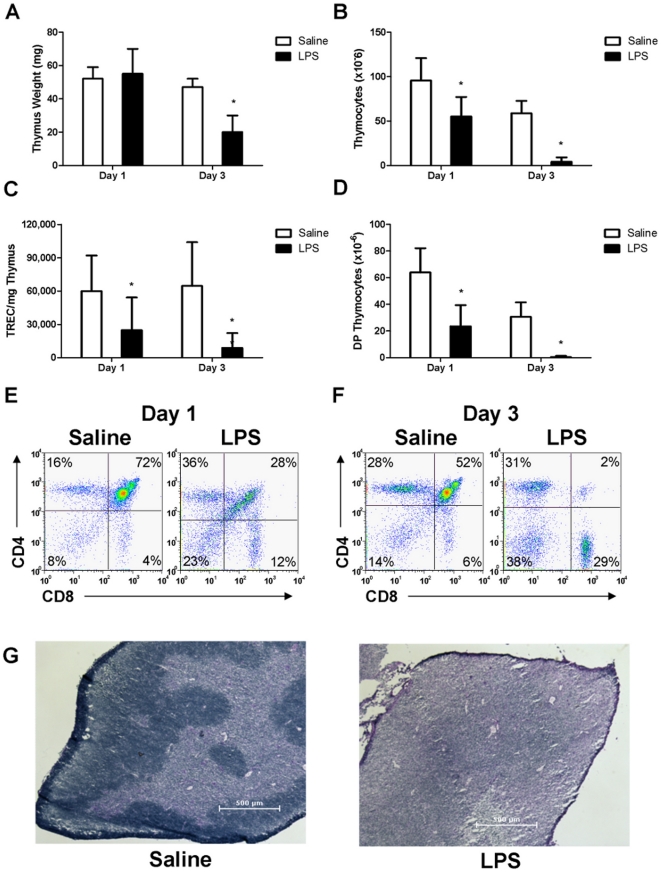
Endotoxin-induced acute thymic atrophy. Female mice were administered either saline or *E. coli* LPS. (A) Thymus weight, (B) Total number of thymocytes, (C) mTREC/mg thymus, (D) Absolute number of DP thymocytes. Representative flow cytometry plots of thymocyte phenotype were determined at (E) one and (F) three days post treatment. (G) Representative hematoxylin and eosin staining of thymus tissue four days post LPS challenge. *p<0.05 compared to saline-treated controls (n = 5).

To demonstrate the effects of endotoxin stress on thymus homeostasis, 8–10 week old mice were injected intraperitoneally (IP) with either saline or 100 µg lipopolysaccharide (LPS) and thymic atrophy was assessed. Thymus weight was unchanged by LPS one day post treatment, yet was significantly decreased by LPS three days post challenge (n = 10) (Figure 1A). Total thymus cellularity however was significantly decreased at both one day and three days post LPS challenge (Figure 1B). A molecular measurement for TCRα gene rearrangement was employed to quantify thymopoiesis. Levels of mTREC/mg thymus were significantly decreased at both one and three days post LPS challenge (Figure 1C). Thymocyte subset distribution was significantly altered with LPS treatment after one day, leading to a specific loss of DP thymocytes by three days (Figure 1D–F).

Changes in thymus architecture were assessed with hematoxylin and eosin staining of frozen thymus tissue four days post LPS challenge. Distinct areas of thymus cortex and medulla were visible in thymus tissue from saline-treated mice (Figure 1G, left panel). In contrast, LPS-treated mice have lost morphological cortico-medullary distinctions (Figure 1G, right panel). Taken together, these data indicated that endotoxemia induces acute thymic atrophy which is detectable at the cellular level by 24 hours post-challenge and resulted in decreased cellularity, decreased TCRα gene rearrangement, specific loss of DP thymocytes, and substantial remodeling of thymic architecture.

### Systemic Cytokine Response Induced by Endotoxin Challenge

A systemic inflammatory cytokine cascade is an important component of the host response to endotoxin challenge, which contributes to septic shock and subsequent acute thymic atrophy. LPS challenge induced peak serum levels (pg/mL) of cytokines measured at 1, 2, 4, or 6 hr post treatment versus saline controls (Table 1). Specifically, significant changes were observed in TNFα, IL-3, IL-10, IL-13, MCP-1, MIP-1β, IL-2, IL-12p40, IL-12p70, KC, GM-CSF, MIP-1α, RANTES, IL-1α, IL-1β, IL-5, IL-6, IL-17, Eotaxin and IFN-γ. These cytokine levels resolved to baseline within 12–24 hours (data not shown). Taken together, these data confirm a potent and significant systemic pro-inflammatory cytokine cascade in this model, consistent with previous published work [1], [4], [13].

**Table 1:** Endotoxin-induced peak cytokine levels in mice (n = 5).

		Treatment		
Cytokine	Peak Hr^a^	Saline	LPS	p-value
TNF-α	1	372±371	59,200±15,9	0.0000349
IL-3	2	3.10±4.28	18.6±12.9	0.0338
IL-4	2	1.01±0.372	1.75±0.667	0.0636
IL-10	2	20.1±22.4	572±376	0.0113
IL-13	2	2.04±4.56	72.1±21.8	0.000110
MCP-1	2	0.780±1.74	39,900±19,800	0.00309
MIP-1β	2	7.89±4.88	36,800±8,000	0.0000134
IL-2	4	0.022±0.05	16.4±8.50	0.00259
IL-12p40	4	193±62.9	24,000±5,770	0.0000155
IL-12p70	4	23.1±15.4	726±217	0.0000889
KC	4	7.83±2.09	58,100±34,800	0.00574
GM-CSF	4	0.526±1.18	99.8±15.3	0.000000515
MIP-1α	4	12.0±26.8	23,400±2,320	0.0000000162
Rantes	4	121±47.2	8,070±2,820	0.000234
IL-1α	6	73.0±6.89	274±97.5	0.00174
IL-1β	6	73.6±60.1	386±81.5	0.000126
IL-5	6	18.1±23.1	843±573	0.0123
IL-6	6	77.1±164	14,900±6,080	0.000622
IL-9	6	34.7±12.8	72.7±37.4	0.0643
IL-17	6	36.7±79.6	493±247	0.00439
Eotaxin	6	131±257	9,910±3,860	0.000478
G-CSF	6	18.9±18.4	16,100±19,900	0.155
IFN-γ	6	3.45±3.29	80.2±46.8	0.00638

a Peak Hr: time of cytokine peak level post-treatment (in hours).

### Intrathymic Cytokine Gene Expression During Acute Thymic Atrophy

To investigate if intrathymic production of pro-inflammatory cytokines was present during thymic atrophy, gene expression analysis was performed on excised mouse thymus tissue. Many of the cytokines that were increased significantly in serum after endotoxin challenge also had significantly increased mRNA level within thymus tissue (Table 2). These included transcripts for potent pro-inflammatory cytokines, such as TNFα, IL-12, KC, MIP-1α, MIP-1β, IL-1α, IL-1β, and IL-6. These observations suggested that thymic residents actively respond to endotoxin stress by up-regulating pro-inflammatory cytokine production. Therefore, further transcriptome analysis was performed to identify other differentially expressed genes and intrathymic pathways following endotoxin stress. Microarray analysis revealed more than 11,000 probe sets with significant changes greater than 1.4 fold one day after LPS challenge (Tables S1 and S2). Additional filtering was thus used to analyze this large number of probe sets (i.e. gene ontology and pathway analysis).

**Table 2:** Endotoxin-induced intrathymic cytokine gene expression.

Probeset ID	Cytokine	Fold Change	Adjusted p-value^a^
1419607_at	TNF-α	2.6	0.0071
1450566_at	IL-3	1.1	0.4082
1449864_at	IL-4	−1.1	0.7036
1450330_at	IL-10	5.4	0.0025
1420802_at	IL-13	1.2	0.2939
1420380_at	MCP-1 (CCL2)	9.8	0.0024
1421578_at	MIP-1β (CCL4)	5.9	0.0020
1449990_at	IL-2	1.2	0.2301
1419530_at	IL-12p40	2.0	0.0282
1449497_at	IL-12p40	1.6	0.0980
1425454_a_at	IL-12p35	1.0	0.8441
1419209_at	KC (CXCL1)	71.7	0.0012
1457644_s_at	KC (CXCL1)	26.0	0.0017
1441855_x_at	KC (CXCL1)	3.8	0.0099
1427429_at	GM-CSF (Csf2)	1.8	0.0160
1419561_at	MIP-1α (CCL3)	57.0	0.0012
1418126_at	Rantes (CCL5)	11.8	0.0046
1421473_at	IL-1α	4.4	0.0096
1449399_a_at	IL-1β	3.5	0.0030
1450550_at	IL-5	1.3	0.1738
1450297_at	IL-6	37.5	0.0018
1450565_at	IL-9	1.1	0.3694
1421672_at	IL-17	1.7	0.0164
1417789_at	Eotaxin (CCL11)	1.6	0.0334
1419427_at	G-CSF (Csf3)	24.7	0.0028
1425947_at	IFN-γ	1.1	0.5898

a p-values adjusted using Benjamini-Hochberg false discovery rate correction.

### Gene Ontology (GO) Analysis of Intrathymic Response to Endotoxemia-Induced Stress

Transcriptome analysis of whole thymus during the early stages of acute thymic atrophy revealed significant gene dysregulation induced by endotoxemia. Significantly modulated intrathymic genes were grouped into gene ontology categories (GO categories) based on statistical overrepresentation of GO terms as determined by a conditional hypergeometric test. Table 3 ranks the top ten GO categories identified in this analysis and lists the top 25 differentially expressed genes within each of the top GO categories.

**Table 3:** Significant differentially expressed genes overrepresented in the gene ontology.

Gene ontology category	p-value^a^	#/GO size^b^	Top 25 fold changes per GO category^c^	% genes change^d^	Mean FC^e^
GO:0001775	2.51×10^−11^	123/201	**Bcl11b**, Bcl3, Cblb, **Ccnd3**, Cd274, **Cd8a**, **Crip3**, **Dclre1c**, Fgg	41.5% up	2.25
Cell activation			**Foxp1**, Gadd45g, Gna13, **Hells**, Il10, **Lat**, **Lig4**, **Msh6**, Pawr,	58.5% down	−2.70
			**Pknox1**, **Rab27a**, **Rag1**, **Rag2**, **Rorc**, **Slamf1**, **Sp3**		
GO:0051301	7.79×10^−10^	131/225	**Aurkb**, **Birc5**, **Bub1**, **Ccne2**, **Ccnf**, **Cdc27**, **Cdc2a**, **Cdc6**, **Cdc7**,	11.5% up	2.17
Cell division			**Cdca7**, **Fam33a**, **Fbxo5**, **Hells**, **Kif11**, **Kntc1**, **Lig4**, **Mcm5**,	88.5% down	−2.82
			**Ncapg2**, Nek6, **Nuf2**, **Sept4**, **Smc2**, **Spc25**, **Ube2c**, Zbtb16		
GO:0046649	1.51×10^−9^	101/165	**Adam17**, **Bcl11b**, Bcl3, Cblb, **Ccnd3**, Cd274, **Cd8a**, **Crip3**,	35.6% up	2.22
Lymphocyte activation			**Dclre1c**, **Exo1**, Fcgr2b, **Foxp1**, Gadd45g, **Hells**, Il10, **Lig4**,	64.3% down	−2.74
			**Msh6**, Pawr, **Pknox1**, **Rab27a**, **Rag1**, **Rag2**, **Rorc**, **Slamf1**, **Sp3**		
GO:0000278	6.19×10^−9^	138/245	**Anln**, **Aurkb**, **Birc5**, **Bub1**, **Ccnf**, **Cdc27**, **Cdc2a**, **Cdc6**, **Cenpe**,	16.7% up	2.33
Mitotic cell cycle			**Fam33a**, **Fbxo5**, Foxg1, **Hells**, **Kif11**, **Kntc1**, **Ncapg2**, Nek6,	83.3% down	−2.68
			**Nuf2**, **Nusap1**, Rhou, Slfn1, **Smc2**, **Spc25**, **Stmn1**, **Ube2c**		
GO:0065007	7.48×10^−9^	2109/5114	Adamts1, Aldh1a1, **Cdca7**, Cebpd, Cfb, Ctgf, Cyr61,	49.7% up	2.63
Biological regulation			F3, Ifi204, Igfbp3, Iigp1, Il6, Lox, Mt1, Mt2, Orm1, Osmr,	50.3% down	−2.30
			Pdk4, Pth, Ptx3, Ramp3, Rorc, **Sh2d1a**, Thbs1, Timp1		
GO:0006955	1.23×10^−8^	119/208	**BE136769**, Ccl12, Ccl2, Ccl3, Ccl5, Ccl7, Ccl9, Cd14, Cd300lf,	74.8% up	6.85
Immune response			**Cd8b1**, Clec4e, Csf3, Ctla4, Cxcl1, Cxcl10, Cxcl13, Cxcl2,	25.2% down	−2.77
			Cxcl5, Cxcl9, Gbp2, Gbp3, Lilrb4, Ptx3, Rsad2, Serpina3g		
GO:0006950	5.06×10^−8^	453/979	Ccl12, Ccl2, Ccl3, Ccl5, Cd14, Cfb, Cxcl1, Cxcl10, Cxcl13,	55.8% up	4.81
Response to stress			Cxcl2, Cxcl5, Cxcl9, Defb1, **Esco2**, F3, Ifi204, Il6, Mx1, Orm1,	44.2% down	−2.41
			Rsad2, Saa1, Saa3, Serpina3n, **Sh2d1a**, Thbs1		
GO:0002521	4.07×10^−7^	72/118	**Ada**, **Adam17**, **Bcl11b**, Bcl3, **Cd24a**, **Cd27**, **Cd4**, **Cd8a**, **Dclre1c**,	25.0% up	4.22
Leukocyte differentiation			**Foxp1**, Gadd45g, **Hells**, Il6, Il7r, **Lck**, **Lig4**, **Pknox1**, **Ppp3cb**,	75.0% down	−2.88
			**Ptprc**, **Rag1**, **Rag2**, **Rorc**, **Sash3**, **Satb1**, **Stat5a**		
GO:0048534	4.81×10^−7^	155/296	**Ada**, **Bcl11b**, Bcl3, **Cd27**, Cd300lf, **Cd4**, **Cd8a**, Csf3, Cxcl13,	41.9% up	3.89
Hematop./lymph. organ devel.			Epas1, Gadd45g, **Hells**, Il6, **Lck**, **Lig4**, **Pknox1**, Plscr1, **Rag1**,	58.1% down	−2.18
			**Rag2**, **Rorc**, Sod2, **Sp3**, **Stap1**, Timp1, Zbtb16		
GO:0002376	5.19×10^−7^	137/261	**Bcl11b**, Bcl2a1a, **Ccnb2**, **Ccnd3**, **Crip3**, Epas1, **Hells**, **Hmgb1**,	46.7% up	2.69
Immune system process			Ifi30, Orm1, **Pknox1**, Plscr1, **Rag1**, **Rag2**, **Rorc**, **Runx1**, S100a9,	53.3% down	−2.50
			Selp, **Slamf1**, Sod2, **Sp3**, **Stap1**, Tapbp, Timp1, Zbtb16		

a Overrepresented GO p-value determined by hypergeometric test.

b Number (#) of individual significant genes present in GO category out of number possible in defined category (GO size).

c Based on absolute value of fold change. Down regulated genes in italics.

d Percentage of individual significant genes that grouped to GO category, showing both up- and down- regulated genes.

e Mean fold changes of genes in each GO category, up- and down- regulated genes averaged separately.

Overall, GO category analysis of thymus tissue transcriptome revealed down-regulation of important processes in thymocyte development and up-regulation of inflammatory and wound healing responses. Taken together, these results suggested that intrathymic gene regulation can directly contribute to inflammation and thymic atrophy induced by systemic endotoxin challenge. Below are categorized descriptions of the significant intrathymic gene expression changes induced by systemic endotoxin challenge.

#### GO Analysis of Intrathymic Genes Involved in Lymphocyte Activation and Thymocyte Development

As predicted, steady state mRNA levels of many factors important for thymopoiesis were down-regulated with endotoxin stress. Several transcription factors were down-regulated, including *Bcl11b*, *Rorc*, and *Runx1*. B-cell CLL/lymphoma 11B (*Bcl11b*) is a transcription factor necessary for DP thymocyte development [15], [16], RAR-related orphan receptor C (*Rorc*) is a transcription factor for Th17 cells [17], and runt-related transcription factor 1 (*Runx1*) is a transcription factor necessary for proper thymocyte development [18]. Several genes involved in VDJ recombination were also down-regulated, including *Dclre1c*, *Lig4*, *Rag1*, and *Rag2* (Table 3).

Furthermore, many genes involved in lymphocyte activation and thymocyte differentiation were decreased, including *Adam17*, *Cd4*, *Cd8*, *Cd27*, *Hells*, *Lat*, *Lck*, *Ppp3cb*, *Slamf1*, and *Stat5a* (Table 3). *Adam17* is a lymphocyte extrinsic factor that is essential for normal DN to DP transition during thymopoiesis [16]. *Cd27* is up-regulated at the DN III to DN IV transition, is further up-regulated upon pre-TCR signaling, and contributes to thymocyte development [19]. Helicase Lymphoid Specific (*Hells*) is involved in chromatin remodeling and transcriptional regulation, and is normally highly expressed in the thymus, functioning to promote differentiation, survival, and/or expansion of thymocytes at the transition from the DN to the DP developmental stage [20]. Linker for activation of T cells (*Lat*), signaling lymphocyte activation molecule family member (*Slamf1*) and leukocyte-specific protein tyrosine kinase (*Lck*), are all important in T cell activation [21], [22]. Calcineurin (*Ppp3cb*) is required for positive selection and contributes to a developmental period of ERK hypersensitivity, allowing very weak signals to induce positive selection [23]. STAT5 plays a major role in IL-7-mediated T cell development [24]. Interestingly, while *Stat5* is down-regulated, IL-7R is up-regulated in response to endotoxemia. Other up-regulated genes of note include Casitas B cell lymphoma b (*Cblb*), which negatively regulates T cell antigen receptor (TCR) signaling and is important for MHC-dependent CD4 and CD8 thymocyte development [25], and the anti-apoptotic proteins PRKC, apoptosis, WT1, regulator (*Pawr*), which down-regulate the anti-apoptotic protein Bcl2 [26] (Table 3).

#### GO Analysis of Intrathymic Genes Involved in Inflammation

Many of the top intrathymic genes identified in the biological regulation GO category (GO:0065007) are involved in tissue inflammation (Table 3). *Adamts1*, an extracellular metalloproteinase that promotes inflammation [27], was significantly increased. CCAAT/enhancer binding protein (C/EBP), delta (*Cebpd*), an important transcription factor for pro-inflammatory cytokine production in a TLR/MyD88-dependent pathway in macrophages [22] was also increased. Factors involved in the coagulation pathway were up-regulated, including tissue factor (*F3*), and thrombospondin 1 (*Thbs1*). Of note, complement factor B (*Ctb*) and connective tissue growth factor (*Ctgf*) were up-regulated.

Important factors involved in pro-inflammatory cytokine cascades were also increased (GO:0006955, GO:0006950), including the pro-inflammatory cytokine IL-6 and the receptor for the IL-6 cytokine family member oncostatin M (*Osm*). Many chemokines were up-regulated in the thymus after endotoxin stress and were categorized in gene ontologies of immune response and response to stress. These included monocyte chemotactic protein (MCP)-5 (CCL12), MCP-1 (CCL2), macrophage inflammatory protein (MIP)-1 (CCL3), Regulated Upon Activation, Normal T-cell Expressed and Secreted (RANTES/CCL5), MIP-1 delta (CCL9), granulocyte colony stimulating factor 3(G-CSF/Csf3), KC (CXCL1), IP-10 (CXCL10), B lymphocyte chemo-attractant (BLC/CXCL13), MIP-2α (CXCL2), epithelial-derived neutrophil-activating peptide 78 (ENA-78/CXCL5), monokine induced by gamma interferon (MIG/CXCL9). Interestingly, *Cd14*, a co-receptor for TLR4, and cytotoxic T-lymphocyte-associated protein 4 (*Ctla4*) were also up-regulated in response to endotoxin stress in the thymus (Table 3) suggesting active TLR responses and negative regulation of thymocyte signaling.

#### GO Analysis of Intrathymic Anti-Inflammatory Genes

Moreover, factors associated with anti-inflammatory properties were also induced by endotoxemia. Endotoxin challenge induced metallothionein 1 and 2 (*Mt1*, *Mt2*), which play anti-inflammatory roles in protection against heavy metal toxicity and scavenging of free radicals [28]. Additional factors up-regulated include tissue inhibitor of metalloproteinase 1 (*Timp1*) and orosomucoid 1 (*Orm1*). Orosomucoid 1 has been shown to stimulate fibroblast proliferation and contribute to the process of wound healing [29].

### Pathway Analysis of Endotoxin-Induced Intrathymic Gene Expression

To further understand how differentially expressed genes may interact to impact intrathymic pathways, significantly modulated genes and expression levels were analyzed using Genego/Metacore pathway analysis software. Pathway analysis combines mRNA expression levels, ontology categories, and defined pathway information in order to predict cellular processes that are involved in a response. This information was ranked by two complementary methods following pathway analysis. Table 4 outlines the top pathways based on number of significant genes identified within a canonical pathway, thus raising the overall pathway significance (Top statistically significant pathways). Table 5 lists pathways using the second method, based on the standard deviation between differentially expressed genes in a given pathway (Top differentially-affected pathways). In other words, the latter analysis ranks pathways with larger absolute differences between control (saline) and endotoxin (LPS) higher.

**Table 4:** Top statistically significant pathways^a^.

Pathway name	p-value
Cytoskeleton remodeling: Cytoskeleton remodeling	3.75×10^−15^
Cytoskeleton remodeling: TGF, WNT and cytoskeletal remodeling	7.19×10^−15^
Immune response: IL-17 signaling pathways	1.45×10^−13^
Cell cycle: Chromosome condensation in prometaphase	1.15×10^−12^
Immune response: CD28 signaling	1.07×10^−10^
Immune response: Immunological synapse formation	1.11×10^−10^
Cell adhesion: Chemokines and adhesion	1.67×10^−10^
Cell cycle: Regulation of G1/S transition (part 2)	3.09×10^−10^
Immune response: T cell receptor signaling pathway	4.36×10^−10^
Cell cycle: Cell cycle (generic schema)	9.56×10^−10^

a Significance based on number of genes within defined pathways.

**Table 5:** Top differentially-affected pathways^a^.

Pathway name	p-value
Immune response: TLR3 and TLR4 induce TICAM1	3.40×10^−03^
Neurophysiological process: ACM regulation of nerve impulse	2.59×10^−03^
Development: EGFR signaling pathway	1.47×10^−09^
Immune response: CD28 signaling	1.07×10^−10^
Immune response: Regulation of T cell function by CTLA-4	1.18×10^−08^
Immune response: CD40 signaling	5.09×10^−08^
Inhibitory action of Lipoxins and Resolvin E1 on neutrophil functions	8.41×10^−02^
Immune response: MIF-JAB1 signaling	8.24×10^−03^
Ca(2+)-dependent NF-AT signaling in Cardiac Hypertrophy	2.02×10^−02^
Muscle contraction: EDG5-mediated smooth muscle contraction	2.03×10^−02^

a Significance reflects absolute differences (by standard deviation) with respect to saline vs. LPS.

Top statistical categories included pathways involved in cytoskeletal and matrix remodeling, immune response, and cell cycle pathways (Table 4). When identified pathways were sorted by the degree of difference between saline control and endotoxin-challenged animals (differentially-affected), the effect on the immune system, and thymocyte function specifically, was more pronounced (Table 5) as six of the ten pathways refer specifically to immune-related responses. The top differentially-affected pathway analysis revealed altered expression of genes downstream of TLR3 and TLR4 following endotoxin challenge, suggesting that endotoxin is directly influencing thymus gene expression through toll-like receptor (TLR) binding (Table 5 and Figure S1). This analysis demonstrated a direct impact of LPS on thymus tissue itself, rather than LPS mediating thymic involution solely as an indirect result of systemic responses (i.e. systemic inflammation, HPA axis). The critical pathways identified in this analysis, their component genes, and possible role in thymus atrophy and recovery are described below.

#### Cytoskeletal and Extracellular Matrix Pathway Analysis

**Figure 2:**
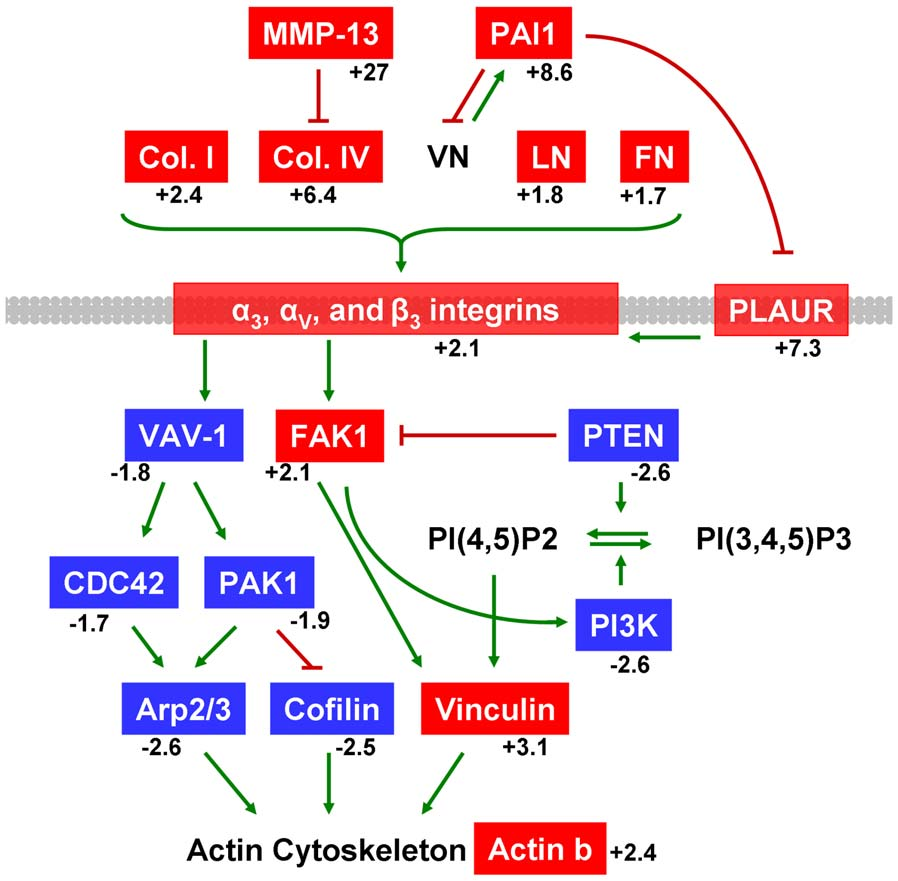
Cytoskeleton and matrix remodeling during acute thymic atrophy. The cytoskeleton remodeling pathway scored highest in Metacore pathway analysis based on number of significant genes (p-value = 3.75×10^−15^). Red genes represent significant increases in mRNA levels and blue genes represent significant decreases in mRNA levels in thymus tissue following LPS treatment. Mean fold changes are given. Green arrows indicate positive influence and red T-bars indicate negative influence. Arrows may represent multiple steps. Plasma membrane is shown in gray. A detailed version of the original Metacore pathway is found as Figure S2. A full legend of all GeneGo pathway map symbols is in Figure S9 or at http://www.genego.com/pdf/MC_legend.pdf.

Figure 2 indicates genes involved with cytoskeletal rearrangement, extracellular matrix (ECM) interactions, and tissue remodeling. This pathway is significantly activated in thymus tissue stressed by endotoxin challenge as revealed by pathway analysis. Several genes encoding members of the fibrinolytic (*Serpine1/PAI1*, *Serping1/C1 inhibitor*, *Plaur*) and matrix metalloprotein (*Mmp13*) proteolytic systems are activated in stressed thymus tissue [30], [31]. *Mmp13* expression was increased 27-fold in endotoxin challenged thymus (Figure 2 and Table S1). In addition, increases in several ECM gene mRNA levels are shown, including collagens (*Col4a1*, *Col4a2*, *Col1a2*, *Col1a1*, *Col4a4*), laminin (*Lamb1*, *Lamc1*), and fibronectin (*Fn1*). Genes for integrins that bind laminin (α3, β1, β4), fibronectin (αV, β1), and collagen (β1) are also upregulated (*Itga3*, *Itgav*, *Itgb1*, *Itgb4*) [32], demonstrating significant tissue remodeling.

In addition to increases in mRNA levels of genes involved in ECM pathways, several cytoskeletal genes are down-regulated intrathymicaly following endotoxin challenge. Decreased expression levels in thymus tissue of *Pak1*, *Vav1*, *Pik3r3*, *Pik3ca*, *Pik3r1*, *Cdc42*, *Ptk2/Fak*, and *Pten* can directly contribute to thymocyte apoptosis through dysregulation of the actin cytoskeleton [33], [34], [35], [36], [37]. Actin (*Actb*) and actin regulators Arp2/3 and cofilin (*Arpc4*, *Cfl1*) also decreased significantly (Figure 2 and Table S2). The observation that the top two significant pathways involved genes for cytoskeleton and ECM remodeling (Table 4 and Figure S2) is consistent with the robust thymus morphological changes observed four days following endotoxin challenge (Figure 1). These intrathymic remodeling pathways can cooperate with those regulating cell cycle and apoptosis to contribute to the loss of thymopoiesis which is characteristic of acute thymic atrophy. Indeed, multiple cell cycle pathways were determined to be significant using Metacore pathway analysis (Table 4). Genes that positively regulate cell cycle progression are down-regulated in thymus following endotoxin challenge (Figure S3).

#### Pathway Analysis of Intrathymic Inflammatory Response Genes

**Figure 3:**
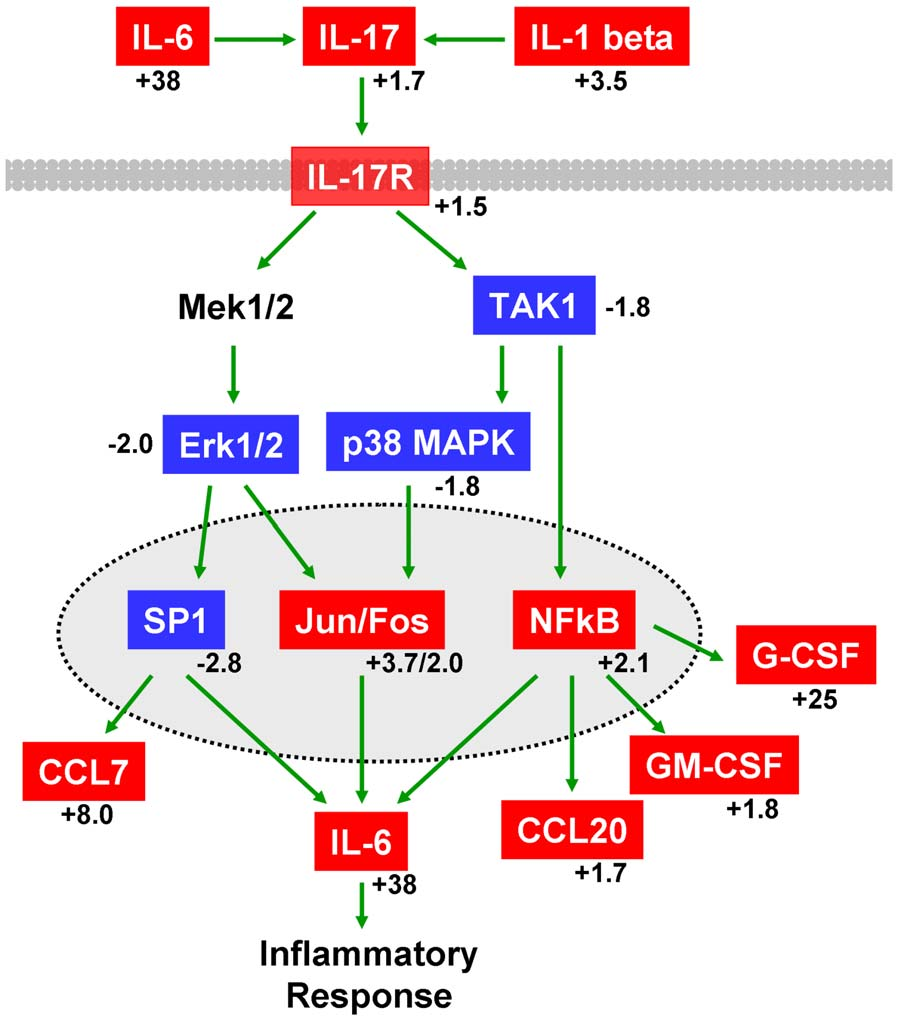
Modulation of IL-17 signaling during acute thymic atrophy. The IL-17 pathway ranked as the third most statistically significant pathway (p-value = 1.45×10^−13^) using Metacore pathway analysis. This pathway includes genes for both secreted pro-inflammatory cytokines and the intracellular response through IL-17R. Red genes represent significant increases in mRNA levels and blue genes represent significant decreases in mRNA levels in thymus tissue following LPS treatment. Mean fold changes are given. Green arrows indicate positive influence. Arrows may represent multiple steps. Plasma membrane is shown in gray. Nuclear membrane is shown as dotted line. A detailed version of the original Metacore pathway is in Figure S4. A full legend of all GeneGo pathway map symbols is in Figure S9 or at http://www.genego.com/pdf/MC_legend.pdf.

IL-6 transcription increases 37.5-fold in thymus tissue from endotoxin challenged mice compared to saline controls (Tables 2 and S1). Additional soluble factors with increased expression included genes for Csf3/G-CSF (24.7 fold), CXCL5 (10.95 fold), CCL12 (10.57 fold), CCL7 (7.96 fold), CXCL3 (4.1 fold), IL-1β (3.5 fold), IL-12β (1.98 fold), Csf2/GM-CSF (1.8 fold), IL-17 (1.73 fold), and CCL20 (1.73 fold). Given that these analyses represented differential gene expression from whole thymus tissue, these results confirmed intrathymic production of inflammatory molecules. Furthermore, several of these cytokines influence expression of IL-17 [38], [39], [40]. Pathway analysis identified the IL-17 signaling pathway as highly significant (Figures 3 and S4), suggesting both intrathymic production of, and response to, pro-inflammatory cytokines and chemokines. Interestingly, the IL-17 signaling pathway contains several down-regulated genes that overlap with other pathways. For example, Map kinases 1, 11, 14, and Mapkkk 7 are also part of the cytoskeletal remodeling pathway (Figure S2), and Sp1 is a transcription factor important for cell cycle (Figure S3). In addition, the IL-1 signaling pathway was highly significant (Figure S5) in this analysis. Taken together, pathway analysis suggested that local production of pro-inflammatory cytokines could directly influence remodeling and cell cycle responses within thymus.

#### Pathway Analysis of Thymocyte Antigen Receptor Signaling

**Figure 4:**
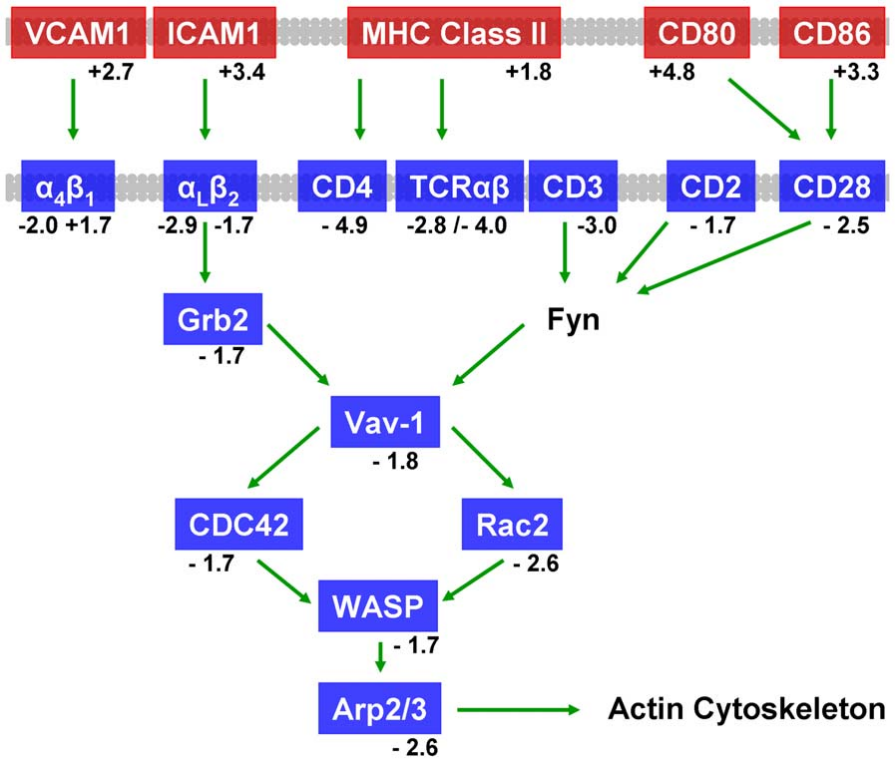
Dysregulation of immunological synapse formation during acute thymic atrophy. The immune synapse pathway ranked sixth in statistical significance (p-value = 1.11×10^−10^) using Metacore analysis. Positive regulators of immune synapse formation and function were decreased following endotoxin stress in thymus. Red genes represent significant increases in mRNA levels and blue genes represent significant decreases in mRNA levels in thymus tissue following LPS treatment. Mean fold changes are given. Green arrows indicate positive influence. Arrows may represent multiple steps. Plasma membranes are shown in gray. A detailed version of the original Metacore pathway is in Figure S6. A full legend of all GeneGo pathway map symbols is in Figure S9 or at http://www.genego.com/pdf/MC_legend.pdf.

**Figure 5:**
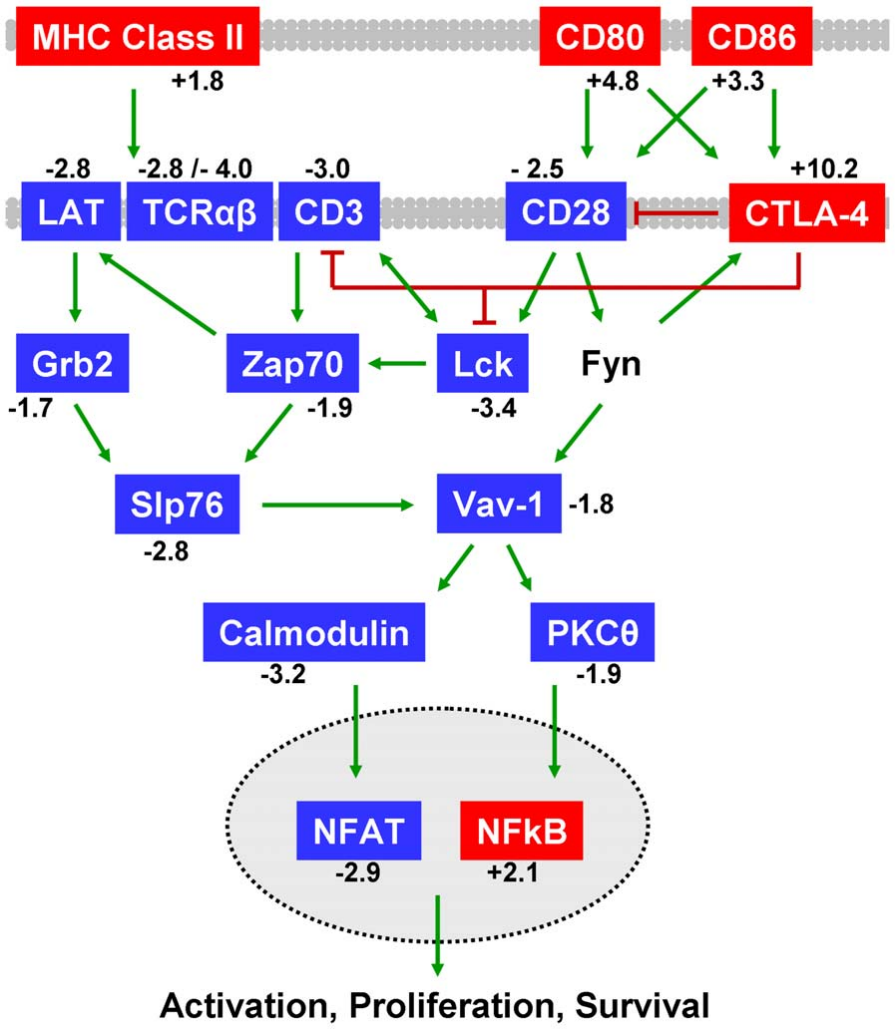
Decreased CD28 signaling and activated CTLA-4 pathways during acute thymic atrophy. The CD28 pathway ranked fifth in significance and fourth by differential expression (p-value = 1.07×10^−10^) using Metacore pathway analysis. Pathway analysis also determined the CTLA-4 pathway to be differentially affected (p-value = 1.18×10^−8^). CTLA-4, CD86 and CD80 mRNA levels were significantly increased while CD28 and TCR signaling-related mRNA levels were decreased. Red genes represent significant increases in mRNA levels and blue genes represent significant decreases in mRNA levels in thymus tissue following LPS treatment. Mean fold changes are given. Green arrows indicate positive influence and red T-bars indicate negative influence. Arrows may represent multiple steps. Plasma membranes are shown in gray. Nuclear membrane is shown as dotted line. A detailed version of the original Metacore pathways are in Figures S7 and S8. A full legend of all GeneGo pathway map symbols is in Figure S9 or at http://www.genego.com/pdf/MC_legend.pdf.

Double positive thymocytes are the most severely affected cell type in thymus, compared to thymic stroma, during endotoxemia, which results in characteristic acute thymic atrophy seen after LPS challenge (Figure 1). Thymocyte functional pathways were therefore examined. Many of the top pathways within the immune response group (Tables 4 and 5) are directly related to T cell signaling, which is important for proper thymocyte development. Pathway analysis of intrathymic gene expression suggested direct cell-cell interactions could play an important role in thymocyte apoptosis in addition to soluble mediators of inflammation. Several pathways involving T cell receptor (TCR) signaling and thymocyte activation were identified as significant by Metacore analysis. Figure 4 depicts a reduction in mRNA levels of genes involved in the thymocyte immunological synapse pathway following LPS challenge. Pathway analysis also predicted a reduction in the TCR/CD28 signaling pathway (Figure 5, Tables 4 and 5). *Cd80*, *Cd86*, *Icam1*, and several H2-A and H2-E (MHC Class II) genes critical for antigen presentation to thymocytes were up-regulated following endotoxin challenge (Figures 4 and 5, Table S1). Interestingly, TCR alpha and beta genes (*Tcra*, *Tcrb-j*), and mRNA for T cell surface molecules CD3 epsilon, CD4, CD28, CD2, and LFA-1 (*Itgal*, *Itgb2*) were significantly lower in the atrophic thymus (Figures 4 and 5, Table S2). These analyses suggested a mechanism for decreased TCR signaling which may contribute to thymocyte apoptosis characteristic of atrophic thymus tissue. Indeed, important signal transduction molecules Lck, Lat, Zap70, Grb2, and PKCθ, as well as down-stream modulators such as calmodulin (*Calm1*, *2*, *and 3*) and NFAT (*Nfatc1* and *Nfatc3*) [41], [42], were all down-regulated (Figure 5 and Table S2). The decreased expression of immune synapse integrin LFA-1 (*Itgal*, *Itgb2*) [43] and key cytoskeletal regulators (*Vav1*, *Wasl*, *Was*, *Cdc42*, *Rac2*) supported an important role for adhesion and cytoskeletal remodeling in TCR signaling [44], [45].

While an abundance of critical TCR signaling-related genes were down-regulated in thymus following endotoxic stress, CTLA-4 was significantly up-regulated (Table S1). The regulation of T cell function by CTLA-4 pathway (Figure 5) was highly significant in this analysis (Table 5). Since CTLA-4 is known to negatively regulate T cell activation in the periphery [46], increased expression in stressed thymus tissue could indicate an active suppression of thymocyte activation leading to apoptosis and subsequent thymic atrophy [47], [48].

## Discussion

Intrathymic mechanisms driving acute thymus atrophy following endotoxemia were investigated in a mouse model. Within 24 hours endotoxin challenge activated a systemic and intrathymic pro-inflammatory cytokine cascade which resulted in losses of thymic cellularity, TCRα gene rearrangement, and DP thymocyte distribution. These characteristic responses to endotoxic stress culminated in significant remodeling of thymus architecture within three to four days post treatment.

A phenotypic, histologic and transcriptome/pathway analysis of murine thymic tissue during the early stages of endotoxemia-induced involution was undertaken in this study to define intrathymic mechanisms that drive this observed acute thymic atrophy. These studies support the hypothesis that multiple key intrathymic pathways are differentially activated during endotoxemia-induced thymic involution and demonstrate for the first time direct activation of thymus tissue by LPS through TLR signaling, local thymus expression of inflammatory cytokines, inhibition of T cell signaling, and induction of wound healing/tissue remodeling.

Whole murine thymus transcriptome analysis one day post endotoxin challenge identified several intrathymic pathways to be modulated during stress-induced acute thymic atrophy, suggesting active involvement of thymic residents in thymopoietic and morphological dysregulation. Findings presented identified up-regulation of genes involved in inflammation and wound healing and down-regulation of genes involved in cell cycle, immune response, and thymocyte activation/signaling during early stages of stress-induced thymic involution. Taken together, these observations suggested that both systemic and direct intrathymic responses to endotoxin challenge concurrently contribute to thymic involution during endotoxemia. These findings are a substantial advancement over current understanding of thymus response to stress and may lead to the development of novel therapeutic approaches to ameliorate immune deficiency associated with stress events.

Transcriptome analysis of whole thymus tissue undergoing acute involution confirmed the active nature of thymus atrophy at the cellular level. Down-regulation of many processes involved in thymocyte proliferation and differentiation were observed, including decreased cell activation and division, mitotic cell cycle dysregulation, and decreased lymphocyte activation and differentiation (Table 3). Important transcription factors (i.e. *Bcl11b*) and factors necessary for lymphocyte development (i.e., *Rag1*, *Rag2*, *Cd4*, *Cd8*) and intracellular signaling molecules (i.e. *Lat*, *Lck*, *Stat5a*) were all down-regulated (Table S2). These data confirmed and provide direct evidence for the rapid decrease in T cell development that occurs following endotoxemia. These observations, however, did not indicate whether the mechanisms driving thymic involution were mediated systemically or intrathymically. Therefore, a complex expression and pathway analysis of genes involved in thymus function and response to stress was undertaken in this study.

A systemic pro-inflammatory cytokine cascade activated by endotoxin-induced stress occurred within the first 24 hours of endotoxemia (Table 1). These observations were consistent with those reported in the literature [4], [6]. There have been no reports however regarding intrathymic production of pro-inflammatory cytokines in response to endotoxin-induced stress. Herein is reported the novel observation that thymus actively up-regulates pro-inflammatory cytokine genes (Table 2). Many of the cytokines up-regulated intrathymically were identical to those produced systemically at the protein level (TNFα, IL-10, MCP-1, MIP-1β, IL-12p40, KC, GM-CSF, MIP-1α, RANTES, IL-1α, IL-1β, IL-6, IL-17, and Eotaxin). This intrathymic up-regulation of cytokine mRNA, many of which are pro-inflammatory, suggested that cells within the thymus itself are producing thymosuppressive factors that likely contribute to thymic atrophy. Gene expression for TNFα (*Tnf*), an important pro-inflammatory cytokine, was up-regulated intrathymically (Table 2), and it has recently been shown that chronic low-level over-expression of TNFα in mice leads to thymic atrophy [49]. Moreover, intrathymic gene expression for IL-6 was significantly up-regulated, which contributes to acute thymic atrophy. Work from this laboratory has previously shown that injection of IL-6, as well as IL-6 family members LIF and OSM, actively induce acute thymic atrophy within three days [50]. Interestingly, the receptor for OSM (*Osmr*) was also up-regulated in response to endotoxemia (Table S1), suggesting that OSM signaling has the potential to be up-regulated as well. Intrathymic regulation of many chemokines was increased (Tables 2, 3, and S1), providing further evidence of a substantial intrathymic inflammatory response. A 23-fold increase in the mRNA of TLR4 co-receptor, CD14, was also seen in these studies (Table S1). Furthermore, TLR-signaling was the top differentially-affected pathway in thymus of endotoxin-challenged mice (Table 5 and Figure S1), strongly suggesting a direct response of cells within the thymus to endotoxin. Activation of a local inflammatory and host response was further supported by the up-regulation of NF-κB (Figure 5 and Table S1). NF-κB can be activated by TLR signaling and by pro-inflammatory cytokines, such as IL-1 and TNFα [51]; both of which were up-regulated intrathymically in response to endotoxin stress. Taken together, these data support the conclusions that thymus tissue directly responds to endotoxin stress by actively up-regulating cytokine and chemokine gene expression, and that these inflammatory mediators may contribute intrathymically to acute thymic atrophy.

While inflammation alone can be damaging, it precedes granulation tissue formation and cytoskeleton/ECM remodeling necessary for wound healing [51]. Many significant changes were detected in the cytoskeleton and ECM remodeling pathway analysis of stressed-thymus tissue (Figure 2). These observations are consistent with the down-stream robust morphological changes observed four days following endotoxin challenge (Figure 1). Several gene expression changes involved in thymic architecture remodeling likely contributed to the degradation of cortico-medullary junctions observed following endotoxin stress; however, many other gene changes suggested the potential for positive tissue remodeling. Transcriptome analysis revealed up-regulation of fibronectin, laminin, and collagen (Figure 2), important ECM components [51]. Up-regulation of these molecules and pathways may be a preparative process for subsequent tissue regeneration and recovery, which has been reported for stress-induced acute thymic atrophy [5].

Other factors that may contribute to thymus tissue recovery include IL-7R and the anti-inflammatory cytokine IL-10. IL-7R was up-regulated, which may indicate a compensatory mechanism, as IL-7 signaling is necessary for thymocyte development [52]. IL-10 was also upregulated and may be important for the resolution of thymic inflammation. The anti-inflammatory activity of IL-10 includes down-regulation of IL-1, IL-6, IL-12, TNFα, and many inflammatory chemokines [53]. Increased thymus transcription of these pro-inflammatory factors may become down-stream targets of IL-10 during resolution of inflammation. Taken together, the involvement of important growth factor receptors and anti-inflammatory mediators, combined with activation of intrathymic wound healing pathways suggested that gene changes necessary for recovery from thymus atrophy are activated upon the onset of involution.

A significant decrease was observed in TCR signaling genes, a pathway necessary for productive thymocyte development [54]. The TCR/CD28 signaling pathway was significantly down-regulated (Figure 5). CTLA-4 mRNA, however, significantly increased following endotoxin challenge (Table S1). Since CTLA-4 is known to negatively regulate T cell activation in the periphery [46], increased expression in the thymus could indicate an active suppression of thymocyte activation leading to apoptosis [47], [48]. This role for CTLA-4 in thymocyte development, however, remains unclear [55], [56], [57]. Cytoskeletal remodeling and adhesion is important for intact TCR signaling [44], [45]. The decreased expression of immune synapse integrin LFA-1 (*Itga1*, *Itgb2*) [43] and key cytoskeletal regulators (*Vav1*, *Wasl*, *Was*, *Cdc42*, *Rac2*) correlated with the observed decrease in TCR signaling machinery, confirming the interaction of cytoskeletal remodeling and TCR signaling pathways (Table S1, Figures 4 and 5). These observations demonstrated how many pathways overlap, with dysregulation of one pathway potentially impacting another.

Data presented herein provide novel evidence that specific gene expression changes are activated in thymus tissue during early stages of stress-induced acute thymus atrophy. Many of these reported gene changes confirmed the process of thymus atrophy, including decreased thymocyte development, activation, and cell cycle progression. These observations correlated with loss of thymus cellularity and decreased thymopoiesis. There were several gene changes, however, that indicated an active intrathymic inflammatory response, in addition to the systemic inflammatory response, and likely contributed to the overall destruction of thymus tissue, induction of thymus atrophy, and loss of thymic output. These observations support the emerging paradigm in which active production of thymosuppressive cytokines/mediators can induce acute thymic atrophy [50], [58]. Therapies that target the depression of intrathymic production of inflammatory factors hold potential to protect the thymus against involution and limit tissue destruction during the important inflammatory response to harmful pathogens or agents. Moreover, it is speculated that early up-regulation of genes involved in wound healing and matrix remodeling in thymus may lay the ground work for thymus recovery. Further investigation is needed to identify recovery mechanisms that are activated at the onset of thymus involution which could be manipulated to hasten recovery of thymus function following stress events and accelerate immune reconstitution. Additional studies are necessary to investigate the interesting possibility that some of these genes may indicate regenerative potential and be exploited for treatment of chronically involuted thymus in the elderly. Taken together, transcriptome analysis of thymus tissue during acute involution demonstrated that the thymus itself is actively responding to stress and contributing with intrathymic inflammatory and regenerative responses.

## Materials and Methods

### Ethics Statement

All mice were housed in a specific pathogen-free environment with 12-hour light/dark cycles at 20–25°C in accordance with Duke University IACUC-approved animal protocols (A152-08-05). All efforts were made to minimize pain and suffering. No human samples were used in this study.

### Animals, Treatments, and Reagents

Female C57BL/6 mice (8–10 weeks old) were purchased from Charles River. *Escherichia coli*-derived lipopolysaccharide (LPS) was purchased from Sigma-Aldrich (L-2880; St. Louis, MO) and reconstituted at 1 mg/mL in PBS. LPS (100 ug) or saline were administered by intraperitoneal injection. Replicate groups of animals were bled at various time points to determine serum cytokine levels prior to euthanasia for tissue harvest (30 minutes to 7 days post treatment). Serum was isolated by centrifugation for 10 min at 3,000×g and transferred to a 96-well round-bottom culture plate and stored at −20°C until thawed for analysis. Mice were euthanized by CO_2_ administration for 10 minutes followed by cervical dislocation. Thymus tissue was removed and weighed. Organs were divided into two halves; one half was placed in a 60-mm tissue culture dish containing 3 mL RPMI 1640 (Invitrogen; Grand Island, NY) with 5% fetal calf serum (tissue medium) and one half was placed into a 1.8 mL cryotube and snap frozen in an ethanol/dry ice bath.

### Cell Isolation and Flow Cytometry

Thymus tissue was teased to a single-cell suspension through a 70 µm cell strainer (BD Labware; Franklin Lakes, NJ) in tissue medium. Thymocytes were centrifuged at 1,500 rpm for 5 minutes and resuspended in 2–5 mL tissue medium for cell counts and immunofluorescent staining. Cell counts were performed in triplicate on a Coulter Z1 Dual threshold cell counter (Coulter; Hialeah, FL) and mean recorded. Total thymus cell counts were extrapolated based on percentage weight of the teased portion of thymus relative to whole thymus weight. Immunofluorescence staining was performed with anti-mouse directly-conjugated monoclonal antibodies: anti-CD3 FITC, anti-CD4 PE, anti-CD8 PerCP-Cy5.5 (BD Biosciences). Cell suspensions were added to PBS Wash (1× PBS+1% bovine serum albumin+0.1% sodium azide) and diluted antibody, incubated for 45 minutes at 4°C, washed and resuspended in PBS Wash containing 0.4% (w/v) paraformaldehyde. Immunophenotype data were acquired on a BD LSRII or BD FACS Canto and analyzed with FlowJo software (TreeStar, Inc.; DHVI Research Flow Cytometry Shared Resource Facility, Durham, NC).

### Quantitative PCR for Mouse Signal Joint TCR Delta Excision Circles (sjTREC)

Total genomic DNA from thymus tissue was extracted using Trizol Reagent (Invitrogen) per manufacturer's protocol. DNA was quantified by spectrophotometry (260 nm). Molecules of mouse *TCRD* sjTREC were quantified by real-time PCR using a standard curve of known number of molecules of mouse (m) TREC, specific primers and fluorescent probe as previously described (21). Briefly, an excess of forward and reverse DNA primers for the mTREC sequence and DNA probe conjugated to a fluorescent dye was added to genomic thymus DNA. Real-time PCR using BioRad iCycler IQ allowed detection and quantification of mTREC numbers per 1 µg DNA in each sample. Numbers of mTREC were normalized to reflect levels per mg thymus tissue.

### Hematoxylin and Eosin Staining of Thymus Tissue

Thymus tissue was removed from mice as described above and embedded in O.C.T. Tissue Tek medium (Sakura). Tissue was frozen in a Histo Bath (Shandon/Lipshaw) and stored at −80°C. Tissue sections (5 µm) were cut on a Cryocut 1800 (Leica Biosystems), placed on Superfrost Plus microslides (VWR International) and stored at −80°C until staining. Sections were fixed for 2 minutes in ice cold acetone (−20°C) and then air dried. Slides were stained with hematoxylin and counterstained with eosin. Analysis was performed with a Nikon Eclipse TE2000-E and NIS Elements 2.0 software (Nikon Instruments Inc) in the DHVI Light Microscopy Shared Resource Facility, Durham, NC.

### Bead-based Multiplex Cytokine Analysis

Serum cytokine levels were determined by multiplex bead-based assays using BioPlex mouse cytokine/chemokine kits according to the manufacturer's protocol (BioRad; Hercules, CA). All bead assay samples were quantified on the BioPlex protein array reader (BioRad) in the DHVI Immune Reconstitution and Biomarker Shared Resource Facility (Durham, NC).

### RNA Extraction and Microarray

Total RNA from snap frozen thymus tissue was extracted using Trizol Reagent (Invitrogen) per manufacturer's protocol and quantified by spectrophotometry (260 nm). RNA was cleaned using an RNeasy Mini Kit per manufacturer's protocol (Qiagen). RNA microarrays were performed by the Duke Microarray Facility in the Duke Institute for Genome Sciences & Policy Department (Durham, NC). Briefly, RNA quality was assessed on an Agilent 2100 Bioanalyzer (Agilent Technologies). Hybridization of total RNA to Affymetrix Mouse genome 430.2 oligonucleotide arrays was performed according to Duke Microarray Facility protocols. Control and LPS treatment data represent experimental replicates.

### Microarray Data Analysis and Statistics

Microarray data analysis was performed using Bioconductor packages under the R statistical language environment [59], [60]. Scanned microarray CEL output files were checked for quality assurance and raw signal intensities were RMA normalized using the affy package [61]. For differential gene expression analysis, the limma package [62] was used for linear model fitting and empirical Bayes methods to determine statistical significance. These genes were then clustered into categories based on over representation of gene ontology terms by hypergeometric test using GOstats package [63]. More detailed procedures can be found at the Bioconductor website (http://www.bioconductor.org). Pathway analysis on statistically significant genes was performed using MetaCore software by GeneGo (St. Joseph, MI). Figures in the text are adaptations based on those created by the MetaCore software. The original MetaCore figures are included in supporting supplemental figures. For animal studies, two-tailed Student's t test was employed to compare the means between data sets.

## Supporting Information

Figure S1 **Activated TLR3 and TLR4 signaling during acute thymic atrophy.** TLR3/TLR4 pathway was the most differentially affected pathway (p = 3.40×10^−3^) when comparing saline-treated to LPS-treated mice. Red data thermometers reflect relative mRNA transcript levels in thymus tissue for control and LPS challenge. EC: extracellular; PM: plasma membrane; IC: intracellular; NU: nuclear. A full legend of all GeneGo pathway map symbols is in Figure S9 or at http://www.genego.com/pdf/MC_legend.pdf.
(TIFF)

Figure S2 **Increased cytoskeleton remodeling during acute thymic atrophy.** The cytoskeleton remodeling pathway scored highest in Metacore pathway analysis based on number of significant genes (p-value = 3.75×10^−15^). Data thermometers reflect relative fold change in mRNA steady-state levels in thymus tissue following LPS challenge. Red thermometers represent significantly increased mRNA levels and blue thermometers represent significantly decreased mRNA levels. EC: extracellular; PM: plasma membrane; IC: intracellular; NU: nuclear. A full legend of all GeneGo pathway map symbols is in Figure S9 or at http://www.genego.com/pdf/MC_legend.pdf.
(TIFF)

Figure S3 **Down-regulation of G1/S cell cycle transition.** The cell cycle transition pathway revealed many genes involved with cell cycle progression to be significantly down-regulated (p = 3.09×10^−10^) in thymus tissue following endotoxin challenge [64]. Data thermometers reflect relative fold change in gene transcript levels in thymus tissue following LPS challenge. Red thermometers represent significantly increased mRNA levels and blue thermometers represent significantly decreased mRNA levels. EC: extracellular; PM: plasma membrane; IC: intracellular; NU: nuclear. A full legend of all GeneGo pathway map symbols is in Figure S9 or at http://www.genego.com/pdf/MC_legend.pdf.
(TIFF)

Figure S4 **Modulation of IL-17 signaling during acute thymic atrophy.** The IL-17 pathway ranked as the third most statistically significant pathway (p-value = 1.45×10^−13^) using Metacore pathway analysis. This pathway includes genes for both secreted pro-inflammatory cytokines and the intracellular response through IL-17R. Data thermometers reflect relative fold change in steady-state mRNA level in thymus tissue following LPS challenge. Red thermometers represent significantly increased mRNA levels and blue thermometers represent significantly decreased mRNA levels. EC: extracellular; PM: plasma membrane; IC: intracellular; NU: nuclear. A full legend of all GeneGo pathway map symbols is in Figure S9 or at http://www.genego.com/pdf/MC_legend.pdf.
(TIFF)

Figure S5 **Modulation of IL-1 signaling pathway during acute thymic atrophy.** Differential gene expression of members of the IL-1 receptor signaling pathway (p = 3.5×10^−9^). Data thermometers reflect relative fold change in gene transcript levels in thymus tissue following LPS challenge. Red thermometers represent significantly increased mRNA levels and blue thermometers represent significantly decreased mRNA levels. EC: extracellular; PM: plasma membrane; IC: intracellular; NU: nuclear. A full legend of all GeneGo pathway map symbols is in Figure S9 or at http://www.genego.com/pdf/MC_legend.pdf.
(TIFF)

Figure S6 **Dysregulation of immunological synapse formation during acute thymic atrophy.** The immune synapse pathway ranked sixth in statistical significance (p-value = 1.11×10^−10^) using Metacore analysis. Positive regulators of immune synapse formation and function were decreased following endotoxin stress in thymus. Data thermometers reflect relative fold change in gene transcript levels in thymus tissue following LPS challenge. Red thermometers represent significantly increased mRNA levels and blue thermometers represent significantly decreased mRNA levels. EC: extracellular; PM: plasma membrane; IC: intracellular; NU: nuclear. A full legend of all GeneGo pathway map symbols is in Figure S9 or at http://www.genego.com/pdf/MC_legend.pdf.
(TIFF)

Figure S7 **Decreased CD28 signaling during acute thymic atrophy.** The CD28 pathway ranked fifth in significance and fourth by differential expression (p-value = 1.07×10^−10^) using Metacore pathway analysis. Genes involved in TCR signaling and thymocyte stimulation via the CD28 pathway are down-regulated in thymus tissue from endotoxin challenged mice. Data thermometers reflect relative fold change in gene transcript levels in thymus tissue following LPS challenge. Red thermometers represent significantly increased mRNA levels and blue thermometers represent significantly decreased mRNA levels. EC: extracellular; PM: plasma membrane; IC: intracellular; NU: nuclear. A full legend of all GeneGo pathway map symbols is in Figure S9 or at http://www.genego.com/pdf/MC_legend.pdf.
(TIFF)

Figure S8 **Activated CTLA-4/CD80/CD86 pathway during acute thymic atrophy.** Pathway analysis of significant genes determined the CTLA-4 pathway to be differentially affected (p-value = 1.18×10^−8^). CTLA-4, CD86 and CD80 were increased while CD28 and TCR-related mRNA levels were decreased. Data thermometers reflect relative fold change in gene transcript levels in thymus tissue following LPS challenge. Red thermometers represent significantly increased mRNA levels and blue thermometers represent significantly decreased mRNA levels. EC: extracellular; PM: plasma membrane; IC: intracellular; NU: nuclear. A full legend of all GeneGo pathway map symbols is in Figure S9 or at http://www.genego.com/pdf/MC_legend.pdf.
(TIFF)

Figure S9 **GeneGo pathway map legend.** Available for download at http://www.genego.com/pdf/MC_legend.pdf.
(PDF)

Table S1 **Significant gene increases within thymus tissue.** Following normalization, microarray data was tested using the *limma* Bioconductor package for linear model fitting and an empirical Bayes method of determination of statistical significance. After false discovery rate was corrected using the Benjamin-Hochberg method, significant gene expression changes (corrected p<0.05) were sorted. Shown are all genes with a mean expression level above 5 and fold changes greater than 1.4.
(XLS)

Table S2 **Significant gene decreases within thymus tissue.** Following normalization, microarray data was tested using the *limma* Bioconductor package for linear model fitting and an empirical Bayes method of determination of statistical significance. After false discovery rate was corrected using the Benjamin-Hochberg method, significant gene expression changes (corrected p<0.05) were sorted. Shown are all genes that decreased more than 1.4 fold. Genes with a mean expression level below 5 are not shown.
(XLS)
