# Supplementary figures and images for: Acute Endotoxin-Induced Thymic Atrophy Is Characterized By Intrathymic Inflammatory and Wound Healing Responses
Source: PLoS One. 2011 Mar 18;6(3):e17940. doi: 10.1371/journal.pone.0017940 (PMC3060875; doi:10.1371/journal.pone.0017940)

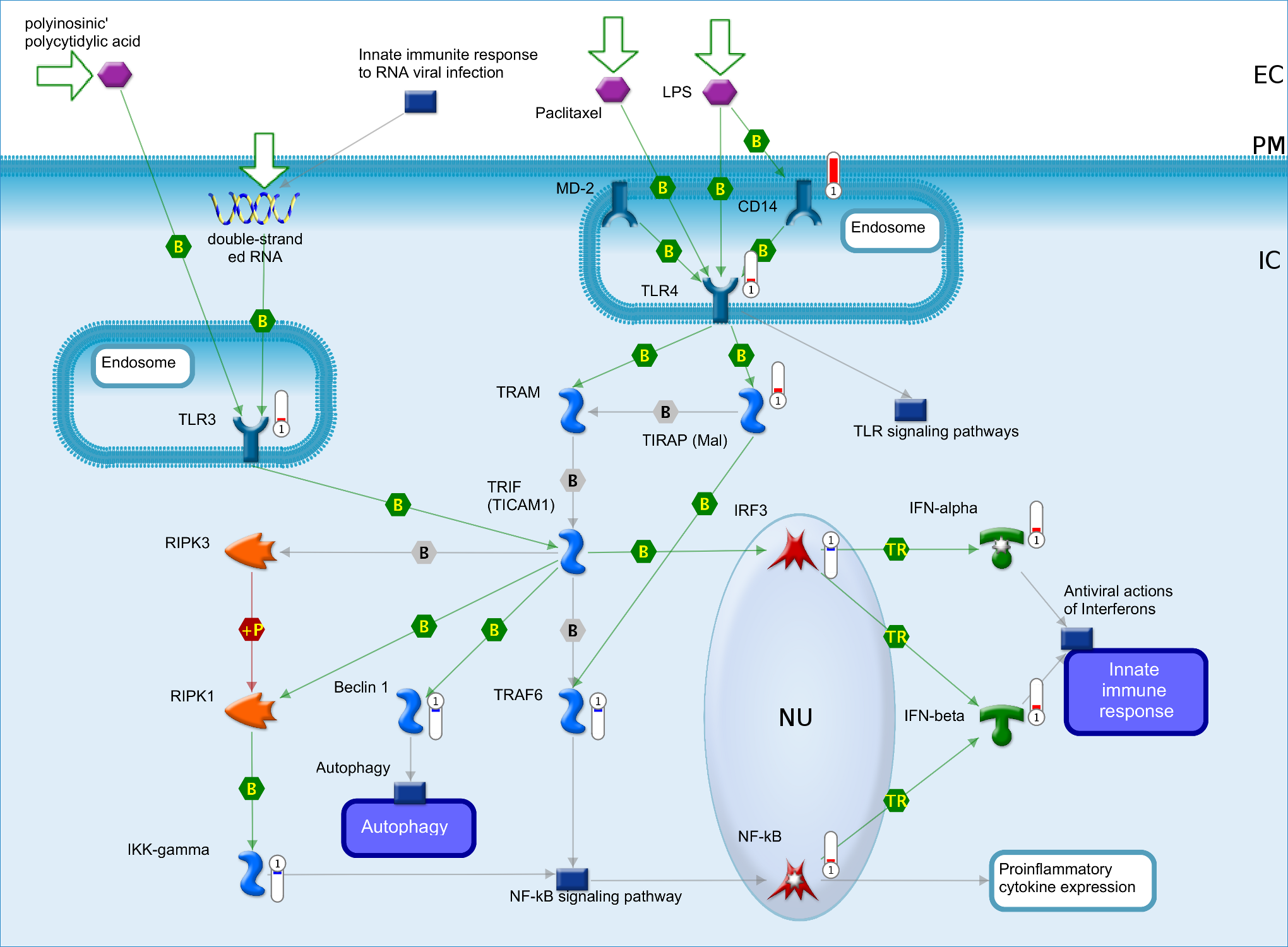

Supplement: Figure S1 — Activated TLR3 and TLR4 signaling during acute thymic atrophy. TLR3/TLR4 pathway was the most differentially affected pathway (p = 3.40×10^−3^) when comparing saline-treated to LPS-treated mice. Red data thermometers reflect relative mRNA transcript levels in thymus tissue for control and LPS challenge. EC: extracellular; PM: plasma membrane; IC: intracellular; NU: nuclear. A full legend of all GeneGo pathway map symbols is in Figure S9 or at http://www.genego.com/pdf/MC_legend.pdf. (TIFF) [file pone.0017940.s001.tif]

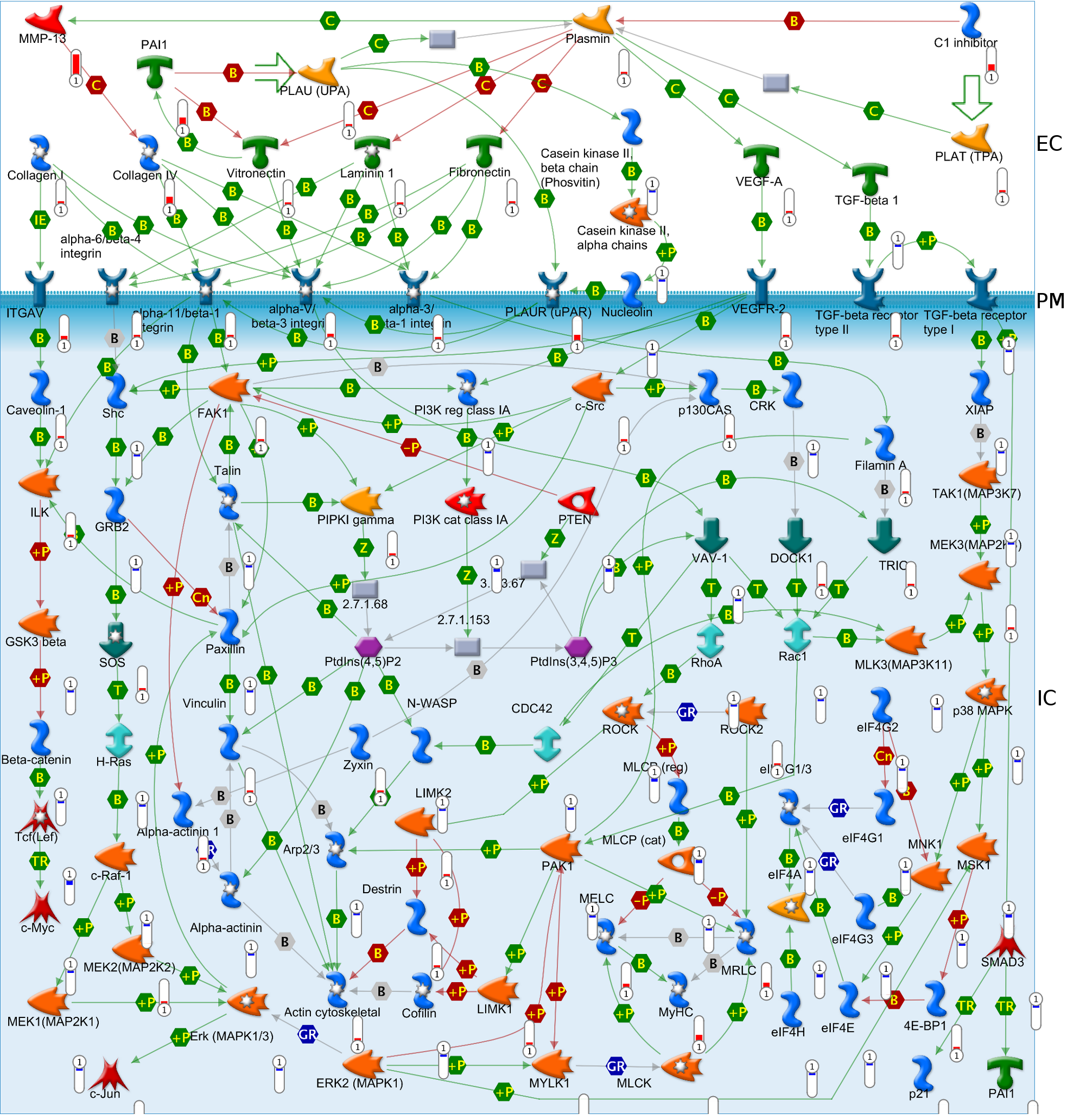

Supplement: Figure S2 — Increased cytoskeleton remodeling during acute thymic atrophy. The cytoskeleton remodeling pathway scored highest in Metacore pathway analysis based on number of significant genes (p-value = 3.75×10^−15^). Data thermometers reflect relative fold change in mRNA steady-state levels in thymus tissue following LPS challenge. Red thermometers represent significantly increased mRNA levels and blue thermometers represent significantly decreased mRNA levels. EC: extracellular; PM: plasma membrane; IC: intracellular; NU: nuclear. A full legend of all GeneGo pathway map symbols is in Figure S9 or at http://www.genego.com/pdf/MC_legend.pdf. (TIFF) [file pone.0017940.s002.tif]

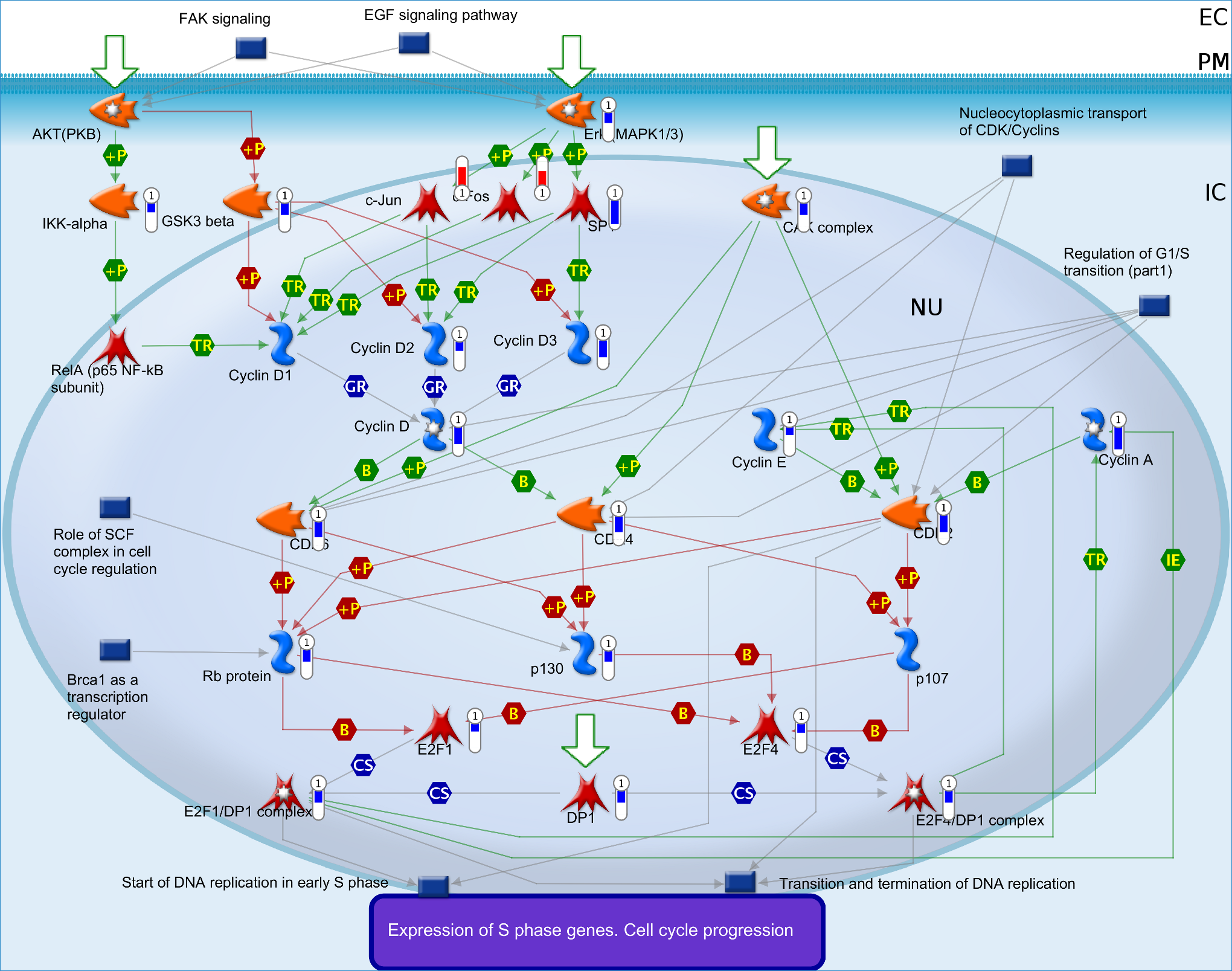

Supplement: Figure S3 — Down-regulation of G1/S cell cycle transition. The cell cycle transition pathway revealed many genes involved with cell cycle progression to be significantly down-regulated (p = 3.09×10^−10^) in thymus tissue following endotoxin challenge [64]. Data thermometers reflect relative fold change in gene transcript levels in thymus tissue following LPS challenge. Red thermometers represent significantly increased mRNA levels and blue thermometers represent significantly decreased mRNA levels. EC: extracellular; PM: plasma membrane; IC: intracellular; NU: nuclear. A full legend of all GeneGo pathway map symbols is in Figure S9 or at http://www.genego.com/pdf/MC_legend.pdf. (TIFF) [file pone.0017940.s003.tif]

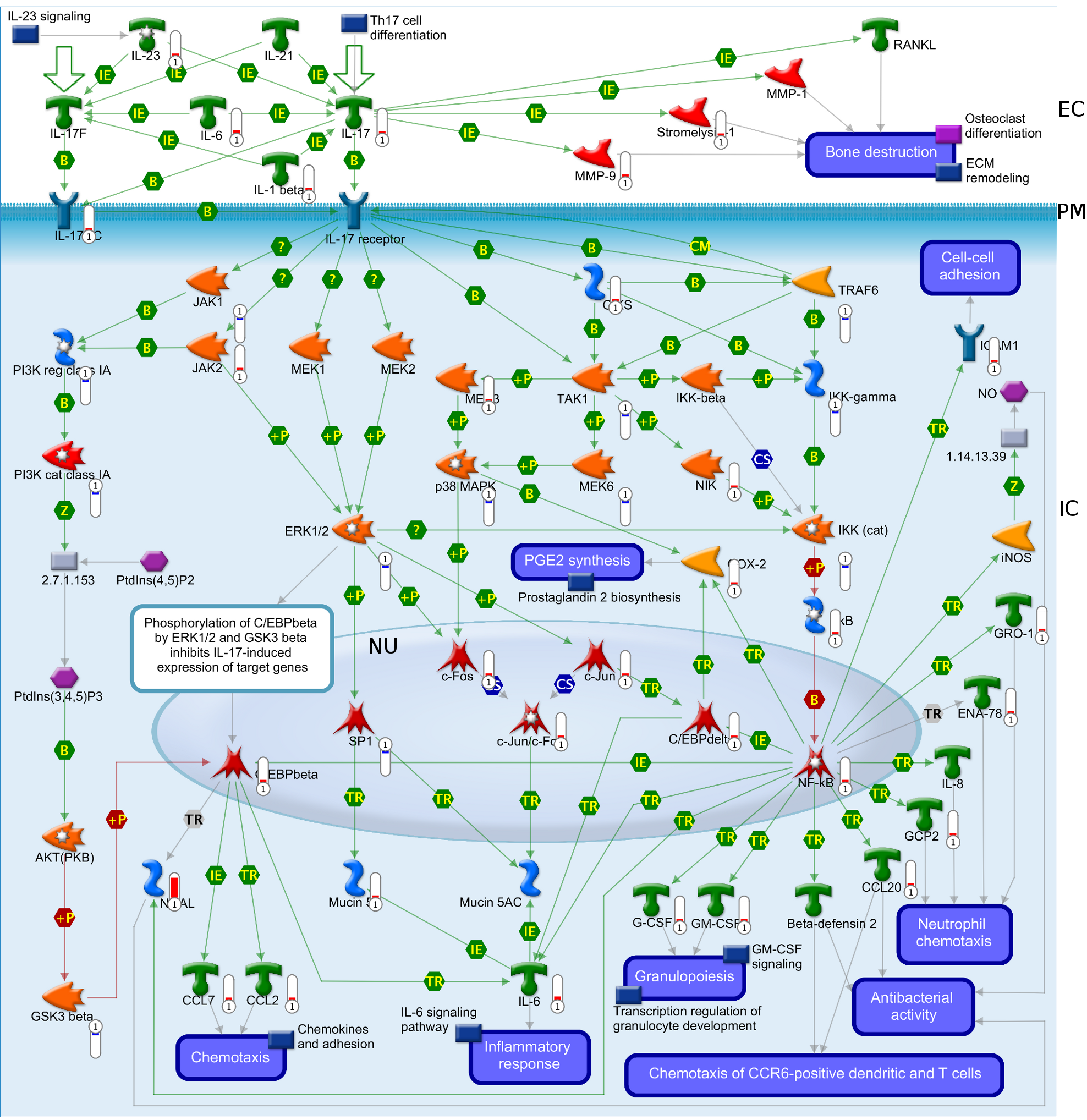

Supplement: Figure S4 — Modulation of IL-17 signaling during acute thymic atrophy. The IL-17 pathway ranked as the third most statistically significant pathway (p-value = 1.45×10^−13^) using Metacore pathway analysis. This pathway includes genes for both secreted pro-inflammatory cytokines and the intracellular response through IL-17R. Data thermometers reflect relative fold change in steady-state mRNA level in thymus tissue following LPS challenge. Red thermometers represent significantly increased mRNA levels and blue thermometers represent significantly decreased mRNA levels. EC: extracellular; PM: plasma membrane; IC: intracellular; NU: nuclear. A full legend of all GeneGo pathway map symbols is in Figure S9 or at http://www.genego.com/pdf/MC_legend.pdf. (TIFF) [file pone.0017940.s004.tif]

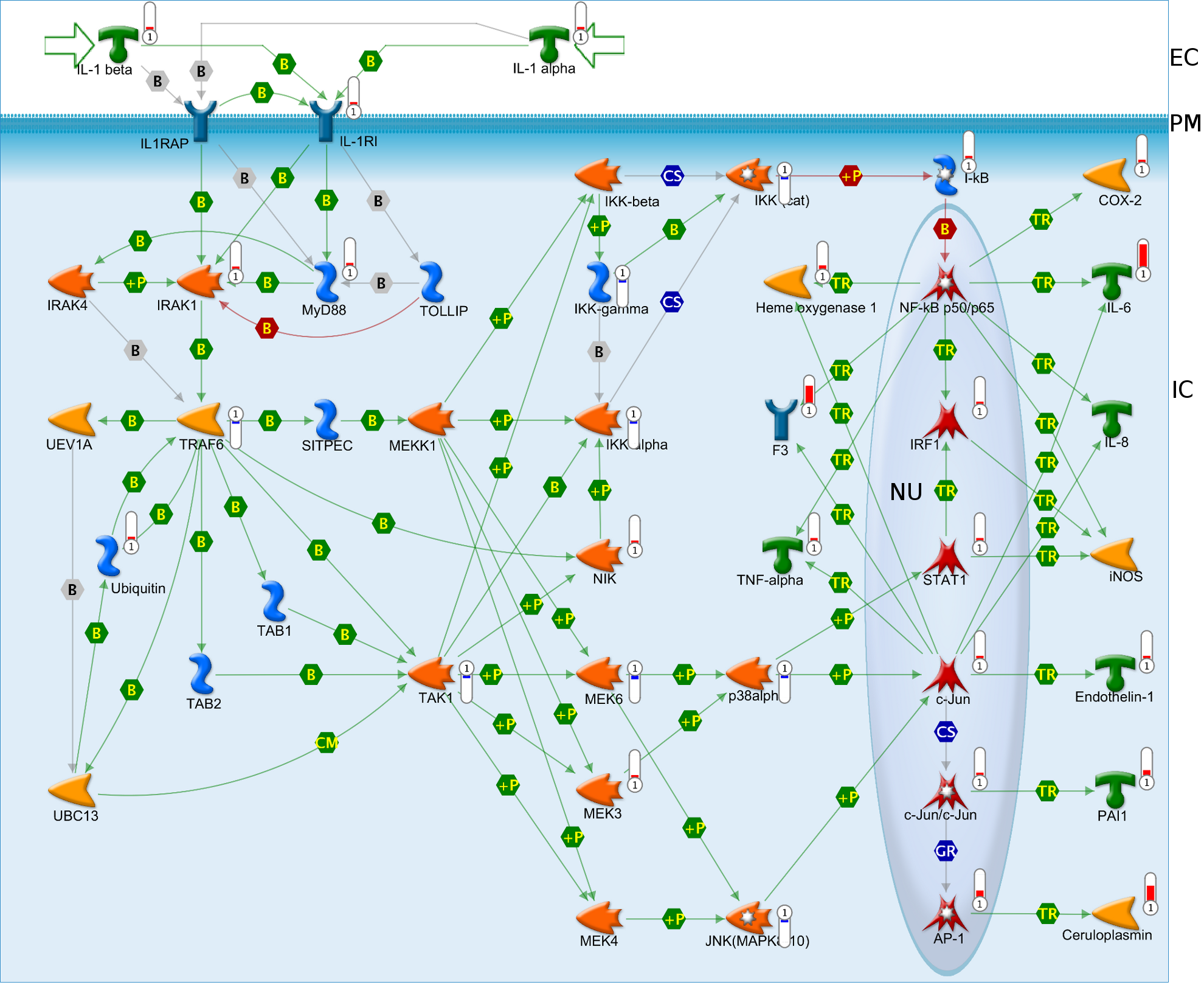

Supplement: Figure S5 — Modulation of IL-1 signaling pathway during acute thymic atrophy. Differential gene expression of members of the IL-1 receptor signaling pathway (p = 3.5×10^−9^). Data thermometers reflect relative fold change in gene transcript levels in thymus tissue following LPS challenge. Red thermometers represent significantly increased mRNA levels and blue thermometers represent significantly decreased mRNA levels. EC: extracellular; PM: plasma membrane; IC: intracellular; NU: nuclear. A full legend of all GeneGo pathway map symbols is in Figure S9 or at http://www.genego.com/pdf/MC_legend.pdf. (TIFF) [file pone.0017940.s005.tif]

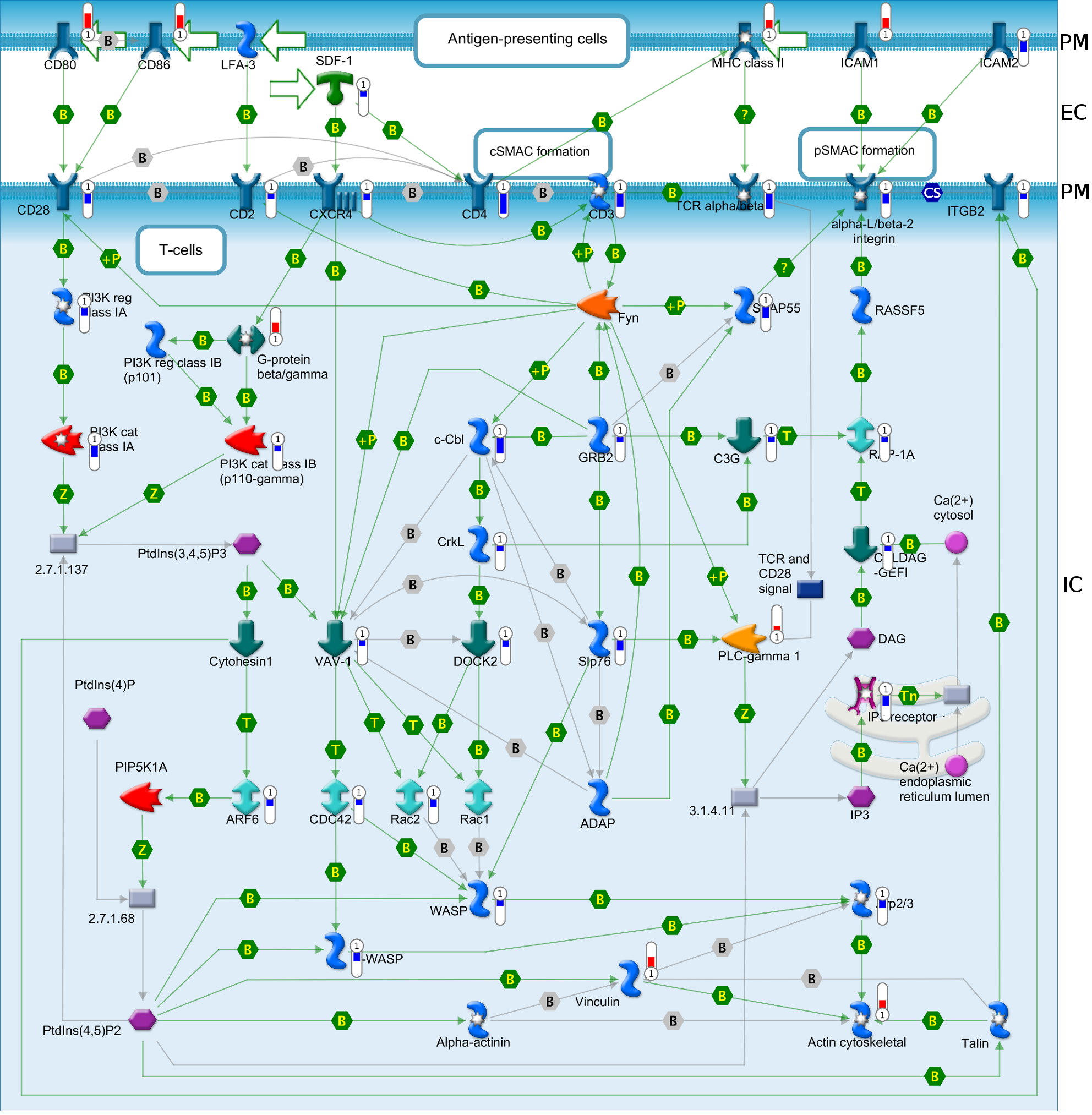

Supplement: Figure S6 — Dysregulation of immunological synapse formation during acute thymic atrophy. The immune synapse pathway ranked sixth in statistical significance (p-value = 1.11×10^−10^) using Metacore analysis. Positive regulators of immune synapse formation and function were decreased following endotoxin stress in thymus. Data thermometers reflect relative fold change in gene transcript levels in thymus tissue following LPS challenge. Red thermometers represent significantly increased mRNA levels and blue thermometers represent significantly decreased mRNA levels. EC: extracellular; PM: plasma membrane; IC: intracellular; NU: nuclear. A full legend of all GeneGo pathway map symbols is in Figure S9 or at http://www.genego.com/pdf/MC_legend.pdf. (TIFF) [file pone.0017940.s006.tif]

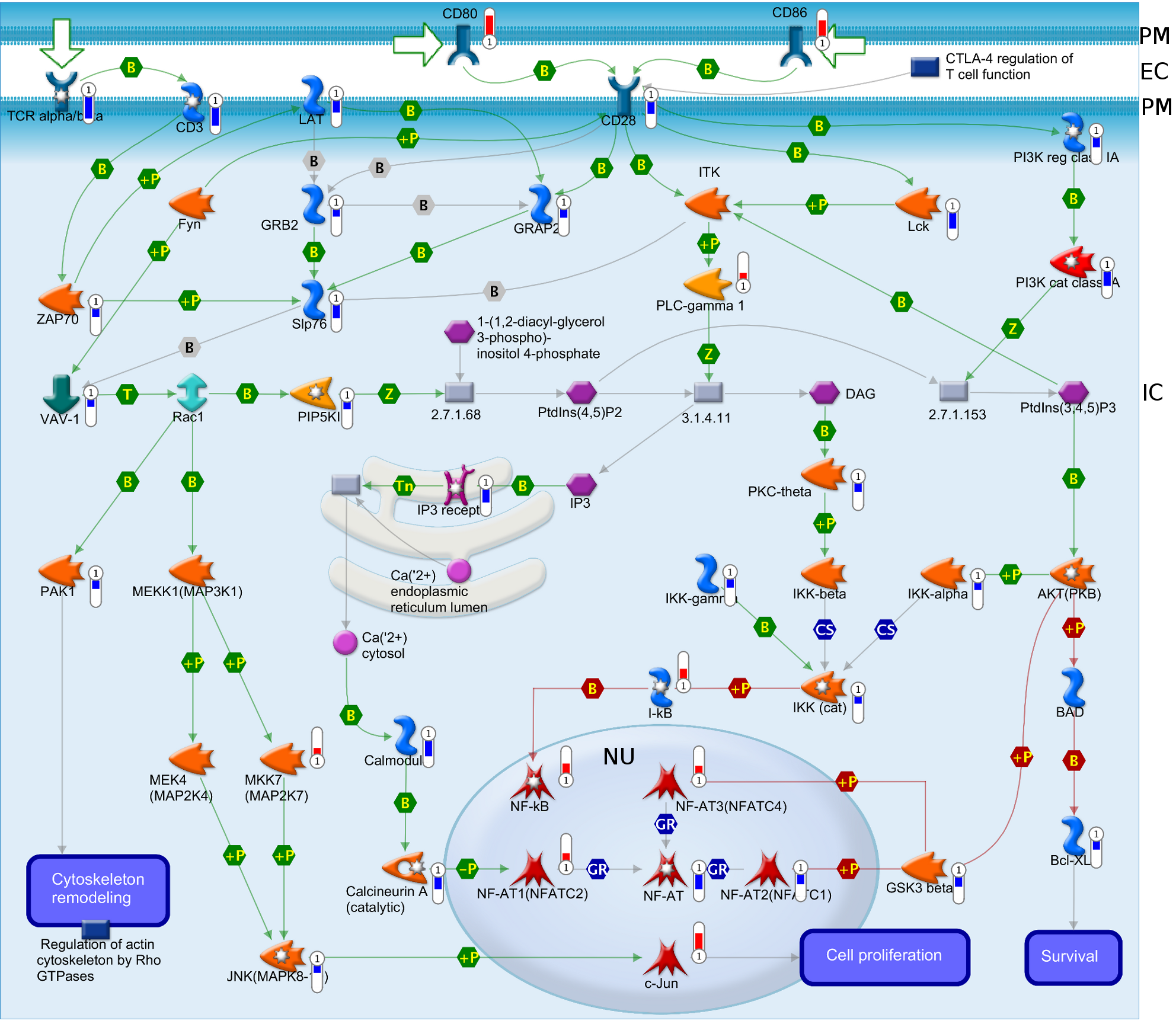

Supplement: Figure S7 — Decreased CD28 signaling during acute thymic atrophy. The CD28 pathway ranked fifth in significance and fourth by differential expression (p-value = 1.07×10^−10^) using Metacore pathway analysis. Genes involved in TCR signaling and thymocyte stimulation via the CD28 pathway are down-regulated in thymus tissue from endotoxin challenged mice. Data thermometers reflect relative fold change in gene transcript levels in thymus tissue following LPS challenge. Red thermometers represent significantly increased mRNA levels and blue thermometers represent significantly decreased mRNA levels. EC: extracellular; PM: plasma membrane; IC: intracellular; NU: nuclear. A full legend of all GeneGo pathway map symbols is in Figure S9 or at http://www.genego.com/pdf/MC_legend.pdf. (TIFF) [file pone.0017940.s007.tif]

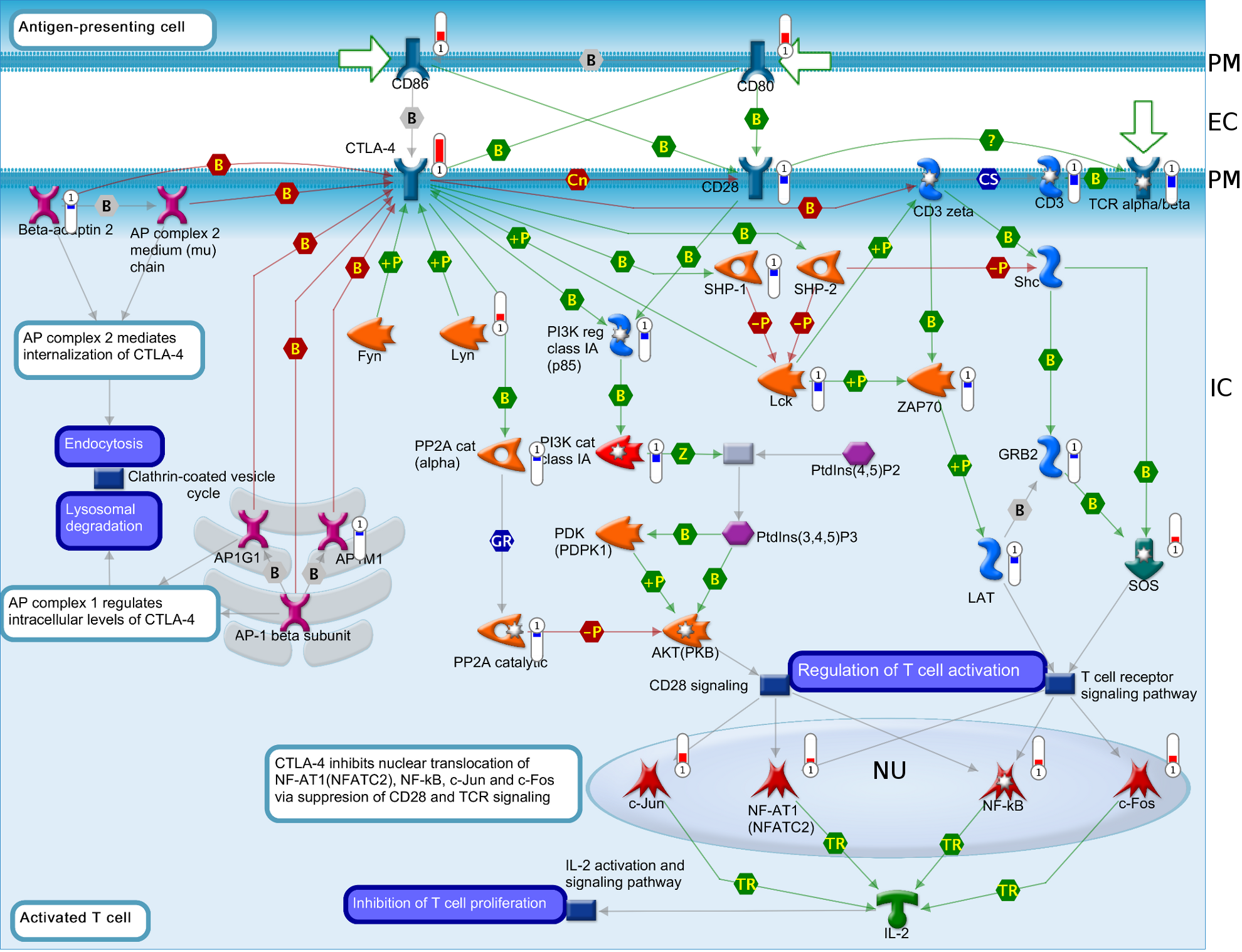

Supplement: Figure S8 — Activated CTLA-4/CD80/CD86 pathway during acute thymic atrophy. Pathway analysis of significant genes determined the CTLA-4 pathway to be differentially affected (p-value = 1.18×10^−8^). CTLA-4, CD86 and CD80 were increased while CD28 and TCR-related mRNA levels were decreased. Data thermometers reflect relative fold change in gene transcript levels in thymus tissue following LPS challenge. Red thermometers represent significantly increased mRNA levels and blue thermometers represent significantly decreased mRNA levels. EC: extracellular; PM: plasma membrane; IC: intracellular; NU: nuclear. A full legend of all GeneGo pathway map symbols is in Figure S9 or at http://www.genego.com/pdf/MC_legend.pdf. (TIFF) [file pone.0017940.s008.tif]
