# Supplementary material for: Acute Endotoxin-Induced Thymic Atrophy Is Characterized By Intrathymic Inflammatory and Wound Healing Responses
Source: PLoS One. 2011 Mar 18;6(3):e17940. doi: 10.1371/journal.pone.0017940 (PMC3060875; doi:10.1371/journal.pone.0017940)
Supplement: Figure S9 — GeneGo pathway map legend. Available for download at http://www.genego.com/pdf/MC_legend.pdf. (PDF) [file pone.0017940.s009.pdf]

MetaCore™ Quick Reference

User Data

Networks / Maps

Up-regulated (+)
Object has user data with positive value

Down-regulated (-)
Object has user data with negative value

Mixed-signal (+/-)
Object has user data with both positive and negative values

Network Objects

Enzymes

Generic enzyme

KINASE
- Generic kinase
- Protein kinase
- Lipid kinase

PHOSPHATASE
- Generic phosphatase
- Protein phosphatase
- Lipid phosphatase

PHOSPHOLIPASE
- Generic phospholipase

PROTEASE
- Generic protease
- Metalloprotease

GTPase
- G-alpha
- RAS - superfamily

Generic Classes

- Receptor ligand
- Transcription factor
- Protein
- Compound
- Predicted metabolite or user's structure
- Inorganic ion
- Reaction
- DNA
- RNA
- Generic binding protein

Channels/Transporters

- Generic channel
- Ligand-gated ion channel
- Voltage-gated ion channel
- Transporter

Receptors

- Generic
- GPCR
- Receptors with kinase activity

G protein Adaptor/Regulators

- G beta/gamma
- Regulators (GDI, GAP, GEF, etc.)

Groups of Objects

A complex or a group
Proteins physically connected into a complex or related as a family

Logical association
Proteins linked by logical relations or physical interactions

Group1
Custom association
Group of collapsed objects chosen by user

Other Marks

Red circle
The links terminated due to a restriction of the number of steps in network expansion.

Blue circle
The links terminated due to network truncation.

Interactions Between Objects

Effects

- Positive / activation
- Negative / inhibition
- Unspecified

Mechanisms

PHYSICAL INTERACTIONS

B — Binding
Compound binds the enzyme or receptor

C — Cleavage
Cleavage of a protein at a specific site yielding distinctive peptide fragments. Proteolytic cleavage can be carried out by both enzymes and compounds

CM — Covalent modifications
Protein activity regulation by covalent binding of a small chemical group to the aminoacids of an active site.

+P — Phosphorylation
Protein activity is altered via addition of a phosphate group

- P — Dephosphorylation
Protein activity is altered via removal of a phosphate group

T — Transformation
Protein activity regulation by binding & hydrolysis of GTP

Tn — Transport
Transport of a protein or a compound between organelles

Z — Catalysis
Catalysis of an enzymatic reaction

Tr — Transcription regulation
Physical binding of a transcription factor to target gene's promoter

M — MicroRNA binding
Regulation of gene expression by binding of microRNA to target mRNA

FUNCTIONAL INTERACTIONS

IE — Influence on expression
Compounds change the expression level of target genes indirectly, for instance by binding to upstream receptors

Cn — Competition
Protein activity regulation by competition at the substrate binding site

? — Unspecified interactions
Mechanism is unknown or/and effect is indirect

PE — Drug-Drug interactions. Pharmacological effect
Drugs change pharmacological effects of other drugs, for instance by competing for drug metabolism enzymes or organic transporters

TE — Drug-Drug interactions. Toxic effect
Drugs change toxic effects of other drugs, for instance by competing for drug metabolism enzymes or organic transporters

LOGICAL RELATIONS

GR — Group relation
Object belongs to a generic group of related objects

CS — Complex subunit
Protein is a subunit of a protein complex

SR — Similarity relation
Chemically similar compounds with chosen Tanimoto similarity score

Links on Networks

Incoming interaction
When the mouse is over object, yellow link indicates direction to object

Outgoing interaction
Cyan link indicates direction **FROM** the object

Interactions from custom list (MetaLink™)

Interaction is in the network
Interaction is represented by a thin solid line and is highlighted in blue

Interaction is in the base, but not in network
Interaction is highlighted in yellow

Interaction is in the network
Interaction is highlighted in magenta

Canonical pathways

Canonical pathway
The link is highlighted in a thick cyan or magenta line

Links on Maps

- Disrupts in disease
- Weakens in disease
- Emerges in disease
- Enhances in disease
- Species specific interactions

Objects on Maps

Localization

- Mitochondria
- EPR
- Golgi
- Nucleus
- Lysosome
- Peroxisome
- Cytoplasm
- Extracellular

Other Map Objects

- Note
- Normal process
- Pathological process
- Normal map
- Disease map
- Species specific object
- Path start

ge ne
(888) 592-3124
(858) 756-7996
www.genego.com
sales@genego.com

© Copyright 2010 GeneGo Inc, all rights reserved worldwide. GeneGo and MetaCore are trademarks of GeneGo Inc.
